# Promising metal-free green heterogeneous catalyst for quinoline synthesis using Brønsted acid functionalized g-C_3_N_4_

**DOI:** 10.1038/s41598-024-72980-1

**Published:** 2024-10-10

**Authors:** Bandarupalli Krishna, Sounak Roy

**Affiliations:** 1 Department of Chemistry, Birla Institute of Technology and Science, Pilani Hyderabad Campus, Hyderabad, 500078 India; 2 Adama India Pvt. Ltd, Genome Valley, Hyderabad, 500078 India; 3 Materials Centre for Sustainable Energy and Environment, Birla Institute of Technology and Science, Pilani Hyderabad Campus, Hyderabad, 500078 India

**Keywords:** Friedländer synthesis, Heterogeneous catalysis, g-C_3_N_4_, Covalent functionalization synthetic modification, Brønsted acid sites, Chemistry, Materials science

## Abstract

Rationally designing distinct acidic and basic sites can greatly enhance performance and deepen our understanding of reaction mechanisms. In our current investigation, we studied the utilization of Brønsted acid sites within layered graphitic carbon nitride (g-C_3_N_4_) for the first time to enhance the rate of the Friedländer synthesis. The structural and surface analyses confirm the effective integration of -COOH and -SO_3_H groups into the g-C_3_N_4_ lattice. The surface-functionalized g-C_3_N_4_-CO-(CH_2_)_3_-SO_3_H exhibits a remarkable acceleration in quinoline formation, surpassing previously mentioned catalysts, and demonstrating notable recyclability under optimized mild reaction conditions. The heightened reaction rate observed over g-C_3_N_4_-CO-(CH_2_)_3_-SO_3_H is attributed to its elevated surface acidity. By probing the Friedländer reaction mechanism through surface characterization, examination of reaction intermediates, and investigation of substrate scope, we elucidate the pivotal role of Brønsted acid sites. This study constitutes a comprehensive exploration of metal-free heterogeneous catalysts for the Friedländer reaction, offering a unique contribution to the field.

**Supplementary Information:**

The online version contains supplementary material available at 10.1038/s41598-024-72980-1.

## Introduction

**Fig. 1:**
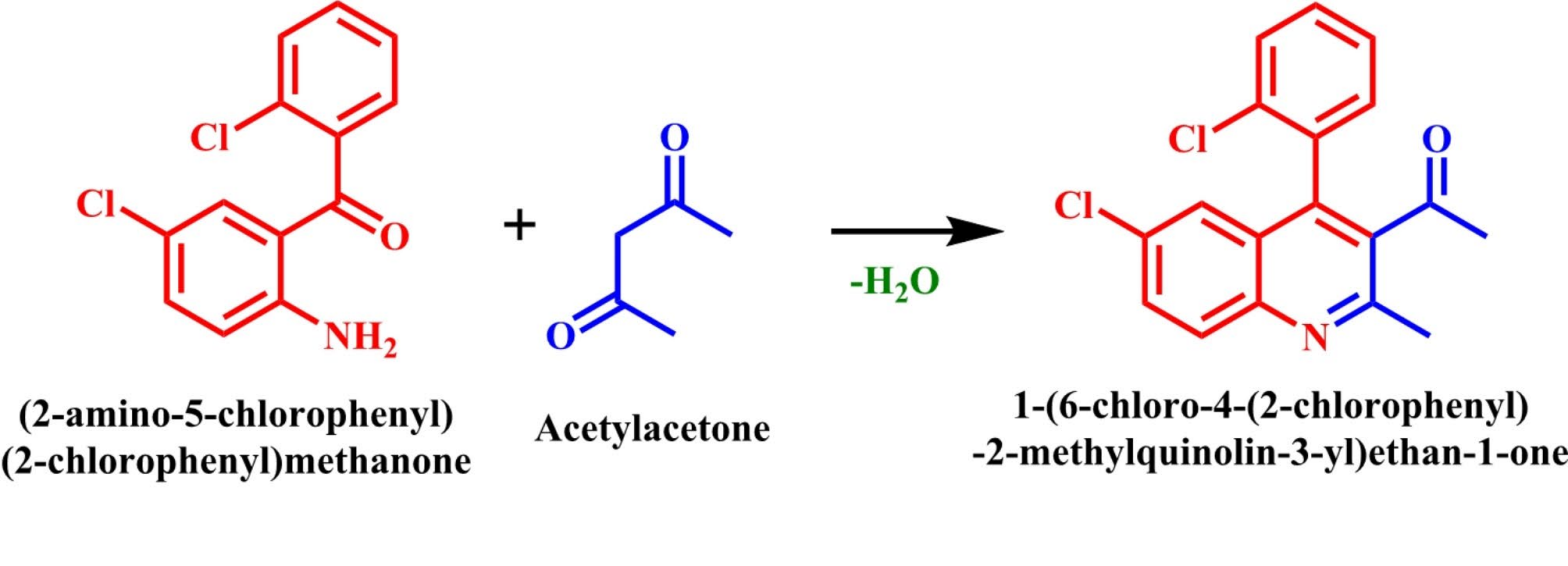
Friedländer synthesis of quinoline derivatives.

Quinoline derivatives, commonly found in natural products exert significant influence in medicinal chemistry due to their diverse biological properties^1–4^. Additionally, their applications are also extended to polymer chemistry^4,5^, sensing^6^ and electrochemistry applications^7,8^. Despite the exploration of numerous methods, the Friedländer synthesis, discovered in 1882, remains one of the most straightforward approaches to generating polysubstituted quinolines (Fig. 1). This procedure typically involves the condensation and cycloaddition of 2-amino aryl ketones and α-methylene carbonyl derivatives, often catalysed by acids.

The Friedländer annulation, which involves the condensation and cyclodehydration of 2-amino aryl ketones and α-methylene carbonyl derivatives, are generally catalysed by classic homogeneous Brønsted acids such as hydrochloric acid, sulfuric acid, *p*-toluenesulfonic acid, and phosphoric acid^9–12^. However, the use of homogeneous acid catalysts has drawbacks, including solvent disposal, and waste generation. Heterogeneous acid catalysts have garnered significant attention in various fields of organic synthesis in recent years^13–15^. Recent studies, therefore have explored a variety of heterogeneous Lewis acids such as CeCl_3_·7H_2_O^16^, FeCl_3_, Mg(ClO_4_)_2_^17^, Bi(OTf)_3_^18^, Y(OTf)_3_^19^, SnCl_2_^20^, and In(OTf)_3_^21^. However, these conventional heterogeneous catalysts encounter issues such as, significant mass transfer resistance, harsh reaction conditions, and susceptibility to the leaching of active sites. Researchers recently focused on developing alternate heterogeneous catalysts for Friedländer synthesis to achieve desired results under milder conditions, and the catalysts, such as as zeolites, metal-organic frameworks, mesoporous silica, zirconia have been explored^22–25^. In our previous work, Brønsted acid functionalized metal-organic framework material MIL-53(Al)-SO_3_H exhibited a very high yield of quinoline under solventless and mild reaction conditions, with a wide substrate scope and good reusability^26,27^.

Very recently, there has been significant interest in the applications of two-dimensional materials in catalysis^28–35^. g-C_3_N_4_ emerges as a particularly significant two-dimensional material, characterized by van der Waals forces between layers composed of tri-s-triazine units interconnected by plane tertiary amino groups^36,37^. g-C_3_N_4_ has attracted attention due to its cost-effectiveness, accessibility, environmental friendliness, and status as a non-metallic organic catalyst^38^. In this work, we present an innovative method for incorporating Brønsted acid sites into g-C_3_N_4_. For the first time, the potential of this metal-free, environmentally friendly green heterogeneous catalyst is studied ameliorating the rate of Friedländer synthesis.

## Experimental section

To synthesize the pristine and functionalized g-C_3_N_4_, the precursors, such as melamine, 1,3-propanesultone, and glutaric anhydride were purchased from TCI Chemicals Pvt. Ltd. The solvents, including N, N-dimethylformamide (DMF), chloroform (CHCl_3_), isopropyl alcohol (IPA), methanol (MeOH), ethanol (EtOH) and toluene were purchased from Merck Specialties Pvt. Ltd. (India). The Nuclear magnetic resonance (NMR) solvents, D_2_O (99.9%), d_6_-CDCl_3_ and d_6_-DMSO were obtained from Sigma-Aldrich. For catalytic reactions,2-aminobenzaldhyde, 2-amino Benzophenone, 2-amino-5-chlorobenzophenone, 2-amino-5-nitrobenzophenone, 2-amino-2’,5-dichlorobenzophenone, acetylacetone, ethyl acetoacetate, cyclohexanone, and other necessary chemicals were procured from Sigma-Aldrich. The synthesized compounds were purified with silica gel (60–120 mesh pore size), ethyl acetate and hexane from Avra Synthesis Pvt. Ltd. (India).

The bulk g-C_3_N_4_ was produced by heating melamine at a temperature of 550 °C for a duration of 4 h, with a heating rate of 2.3 °C per minute. The obtained g-C_3_N_4_ was collected and crushed using a mortar and pestle^29^. For synthesis of g-C_3_N_4_-CO-(CH_2_)_3_-CO_2_H, 300 mg of the as-prepared g-C_3_N_4_ was suspended in 5 mL of toluene, followed by the addition of 600 mg of glutaric anhydride into the mixture. The mixture was refluxed under magnetic stirring for 24 h, and the sample was filtered off and washed three times with toluene, EtOH, and H_2_O to remove the unreacted glutaric anhydride. The functionalized g-C_3_N_4_-CO-(CH_2_)_3_-CO_2_H was dried in a vacuum oven at 80 °C overnight, yielding 310 mg of product. To synthesize g-C_3_N_4_-CO-(CH_2_)_3_-SO_3_H, the as-prepared g-C_3_N_4_ (300 mg) was suspended in 5 mL of toluene within a 50 mL round-bottom flask. Following this, 600 mg of 1,3-propanesultone was introduced into the mixture. The mixture was refluxed using magnetic stirring for 24 h, and filtered and washed the sample three times with toluene, EtOH, and H_2_O to remove any unreacted 1,3-propanesultone. The functionalized g-C_3_N_4_-CO-(CH_2_)_3_-SO_3_H was dried in a vacuum oven at 80 °C overnight, yielding 380 mg of product.

The X-ray diffraction (XRD) studies and phase analysis on the synthesized materials were conducted using a Rigaku Ultima IV diffractometer with Cu Kα radiation (λ = 1.5418 Å) at a scan rate of 1 ^o^/min and step size of 0.01 ^o^. The average crystalline diameters (D) were determined using Scherrer’s formula: $\:\text{D}=\frac{0.9{\uplambda\:}}{{\upbeta\:}\text{C}\text{O}\text{S}{\uptheta\:}}$, where 0.9 is the shape factor, β is the full-width at half-maxima, λ is the X-ray wavelength, and θ is the corresponding angle. Fourier Transform Infrared Spectroscopy (FTIR) spectra of the synthesized samples were obtained using a JASCO 4200 spectrometer with a resolution of 4 cm^−1^. The thermal decomposition of the catalysts was evaluated by thermogravimetric analysis (TGA) using a Shimadzu DTG-60 under an N_2_ atmosphere, with a scan rate of 10 ^o^C/min within the temperature range of 30–600 ^o^C. To determine the surface morphology of the samples, a Field Emission Scanning Electron Microscope (FE-SEM, FEI-Apero S) was utilized. X-ray photoelectron spectra (XPS) were collected using a Thermo Scientific K-ALPHA surface analysis spectrometer with Kα radiation (1486.6 eV) to examine the chemical bonding and surface elemental state of the as-synthesized catalyst. The binding energy of the other elements was adjusted with reference to C 1s of 284.8 eV and elemental analysis was done by using CHNS analysis to find the ratio of sulphur on functionalized g-C_3_N_4_.

To prepare the target quinoline derivatives, 2-aminoaryl ketones (1.0 mmol) and α-methylene carbonyl derivatives (1.2 mmol) were dissolved in a solvent, or without a solvent. Pristine and modified g-C_3_N_4_ catalysts, containing 10 wt% relative to 2-aminoaryl ketone, were added to the reactants. The reaction mixture was stirred using a magnetic stirrer for 4–6 h, while the temperature was varied from 25 to 100 °C. The progress of the reaction was monitored by TLC, using a mobile phase of 20% ethyl acetate in hexane and quantitative NMR using 1,3,5 trimethoxy benzene as internal standard. After the reaction was completed, the mixture was cooled, and the solid catalyst was separated by centrifugation using dichloromethane. The structure was analysed by^1^H-NMR using an AV NEO 400 MHz (Bruker) and LC-MS using a Shimadzu-8040. The recovered catalyst was tested for recyclability for up to five cycles. The reaction conditions were optimized by varying the catalyst loading, reaction temperature, time, solvents, and substrate scope.

## Results and discussion

### Structure and surface properties

**Fig. 2:**
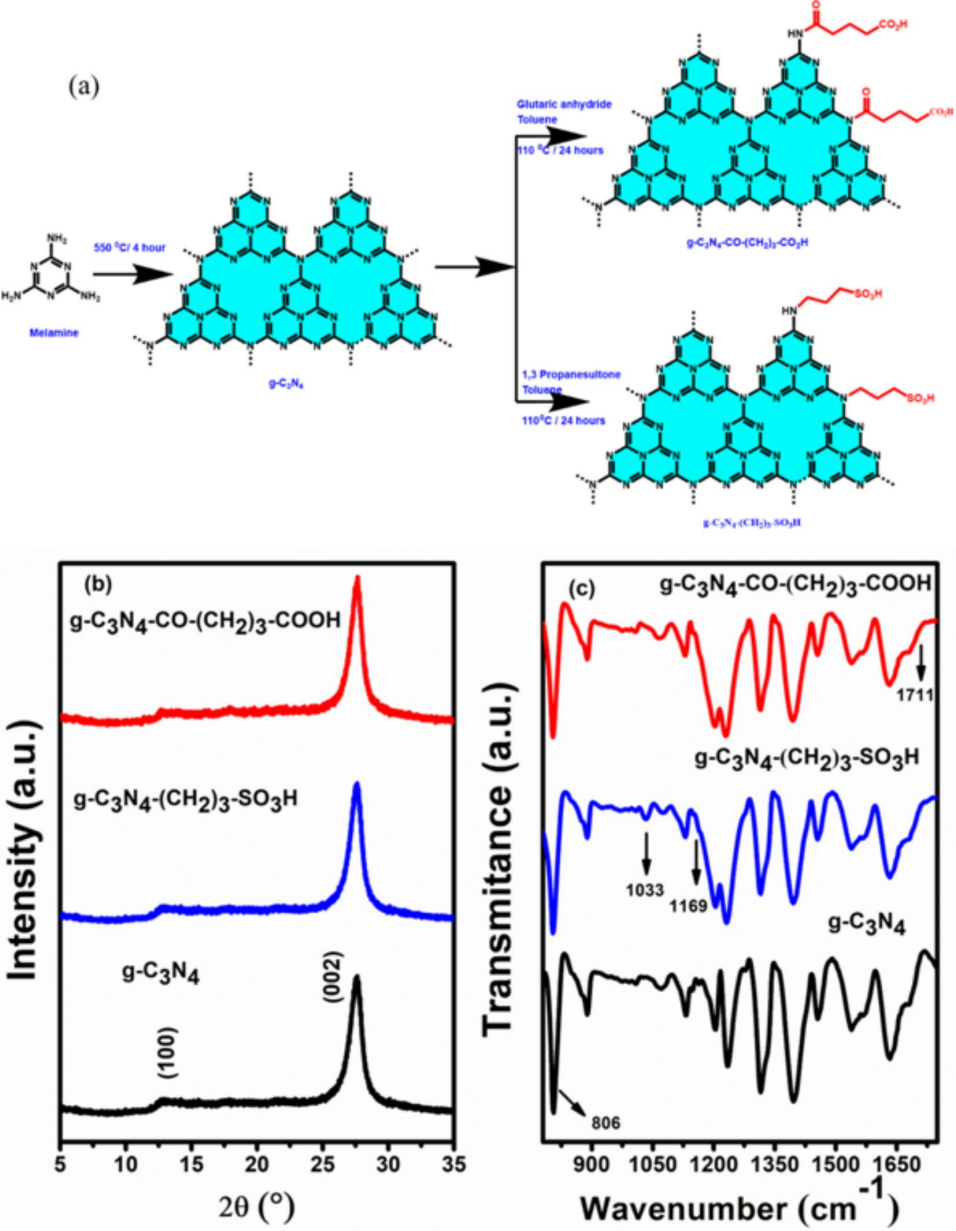
(**a**) Synthesis strategy of the pristine g-C_3_N_4_ and the Brønsted acid functionalized g-C_3_N_4_-CO-(CH_2_)_3_-CO_2_H and g-C_3_N_4_-(CH_2_)_3_- SO_3_H, (**b**) powder XRD patterns, and (**c**) FT-IR spectra of the pristine g-C_3_N_4_ and functionalized g-C_3_N_4_-CO-(CH_2_)_3_-CO_2_H & g-C_3_N_4_-(CH_2_)_3_- SO_3_H.

The successful synthesis of pristine g-C_3_N_4_ and functionalized g-C_3_N_4_-CO-(CH_2_)_3_-CO_2_H and g-C_3_N_4_-CO-(CH_2_)_3_-SO_3_H was confirmed through powder X-ray diffraction. g-C_3_N_4_ was synthesized from melamine, followed by a reaction with glutaric anhydride and 1,3-propanesultone to yield crystalline g-C_3_N_4_-CO-(CH_2_)_3_-CO_2_H and g-C_3_N_4_-(CH_2_)_3_-SO_3_H, as shown in Fig. 2a. In Fig. 2(b), data reveal distinct diffraction peaks from pristine g-C_3_N_4_ at 2θ = 12.4° and 27.6°, corresponding to the (100) and (002) planes, respectively. The (100) plane signifies the in-plane structural^39^ packing of tri-s-triazine or heptazine units, with an interlayer distance of 0.675 nm, while the (002) plane corresponds to the stacked conjugated aromatic rings between layers, with an interlayer spacing of 0.326 nm^29,39–41^. The powder XRD patterns of the Brønsted acid-functionalized g-C_3_N_4_-CO-(CH_2_)_3_-CO_2_H and g-C_3_N_4_-CO-(CH_2_)_3_-SO_3_H catalysts exhibit similar peaks to g-C_3_N_4_, indicating remarkable structural consistency after functionalization. The nanocrystalline diameters of both pristine and functionalized g-C_3_N_4_ were calculated using Scherer’s formula, resulting in an average size range of 4 to 5 nm. The FTIR spectra in Fig. 2(c), further confirm the chemical structures of the g-C_3_N_4_ and acid functionalized g-C_3_N_4_. The functionalized g-C_3_N_4_ exhibited major characteristic IR spectra similar to bulk g-C_3_N_4_. The characteristic peaks in the region 1200–1650 cm^− 1^ correspond to N–(C)_3_ and C–N = C stretching vibrations^42,43^, confirmed that the basic functional units are retrained after functionalization on g-C_3_N_4_. The sharp peak at 810 cm^− 1^ is attributed to tri-s-triazine ring vibrations^44,45^. In g-C_3_N_4_-(CH_2_)_3_-SO_3_H, the surface functionalization was further evidenced by the characteristic peaks at 1169 cm^− 1^ and 1033 cm^− 1^ ascribed to asymmetric and symmetric stretching of S = O groups, respectively^46^. The band at 1711 cm^− 1^ present only in g-C_3_N_4_-CO-(CH_2_)_3_-CO_2_H is due to the stretching frequency of C = O in the free carboxylic group. The powder XRD and the FTIR studies confirmed the successful synthesis of bulk g-C_3_N_4_ and their functionalized derivatives. From the CHNS analysis (Table S1) we determined that 20% of g-C_3_N_4_ has been functionalized with -SO_3_H.

**Fig. 3:**
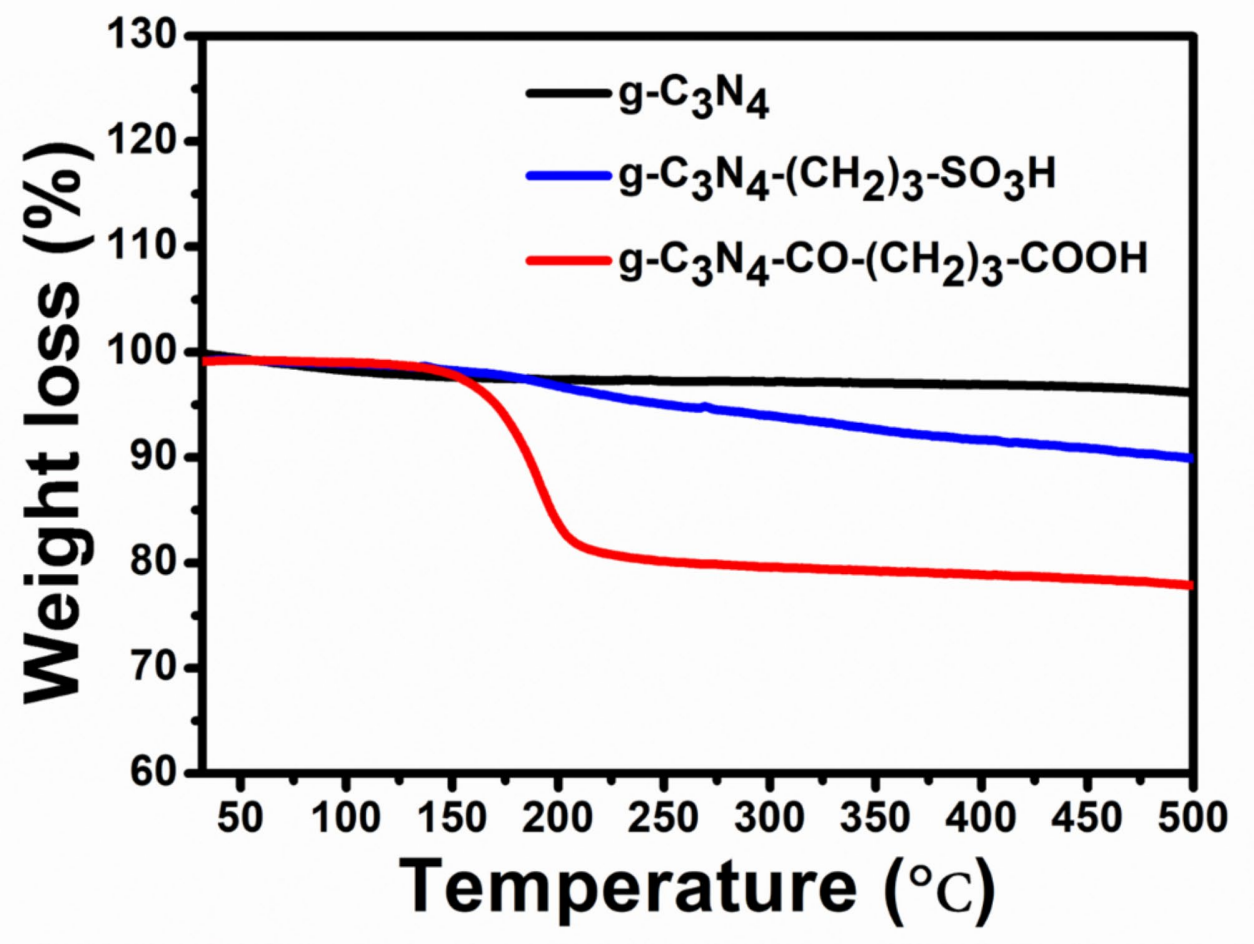
TGA data of pristine and its functionalized g-C_3_N_4_.

The thermogravimetric analysis was conducted up to 500 ^o^C to evaluate the thermal stability of pristine and functionalized g-C_3_N_4_ catalysts. The TGA results displayed in Fig. 3 exhibit a minor weight loss on the functionalized materials compared to pristine g-C_3_N_4_, attributed to the presence of surface functionality. The g-C_3_N_4_ bulk catalyst exhibited remarkable stability within the experimental temperature window with a minimal weight loss of 3.0% due to the desorption of physisorbed surface bound species. The g-C_3_N_4_-CO-(CH_2_)_3_-CO_2_H exhibited ~ 20% weight loss starting at 150 °C, whereas g-C_3_N_4_-(CH_2_)_3_-SO_3_H exhibited a total weight loss of 10%. It is evident from the TGA studies that the stability of functionalized g-C_3_N_4_-(CH_2_)_3_-SO_3_H is superior to g-C_3_N_4_-CO-(CH_2_)_3_-CO_2_H^44^. The higher stability of g-C_3_N_4_-CO-(CH_2_)_3_-CO_2_H could be attributed to the strong N-C bond with respect to the amide type bond in g-C_3_N_4_-CO-(CH_2_)_3_-CO_2_H^47,48^. Additionally, the thermal stability exhibited by the functionalized catalysts is adequate for conducting the Friedländer synthesis reaction at elevated temperatures.

**Fig. 4:**
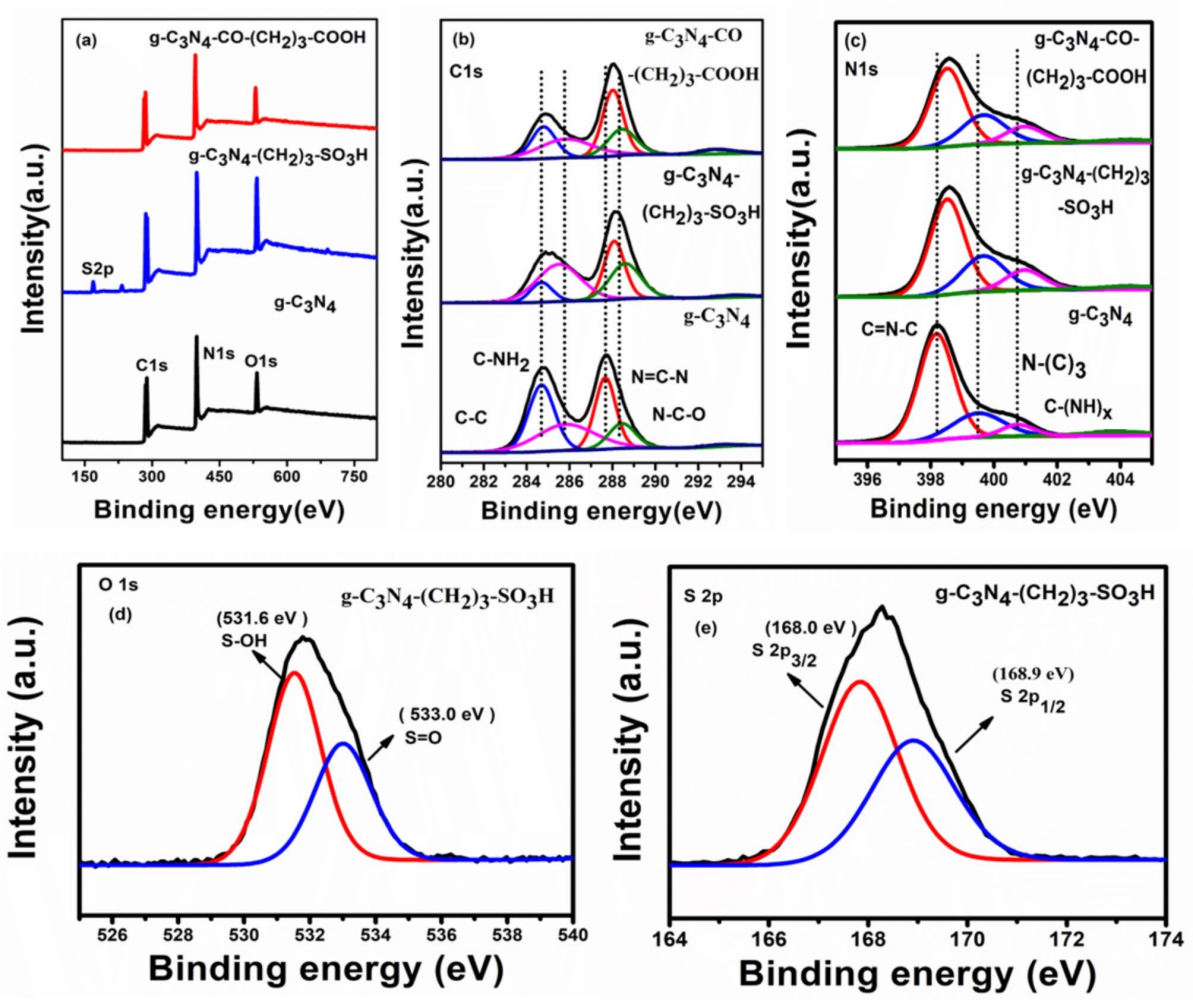
(**a**) XPS survey spectra, High resolution core level spectra of (**b**) C 1s, (**c**) N ls, (**d**) O ls and (**e**) S 2p.

X-ray photoelectron spectroscopy was used to analyse the elemental chemical states of the composite of g-C_3_N_4_ and functionalized g-C_3_N_4_ catalysts (Fig. 4). The XPS survey spectra of the materials in Fig. 4a demonstrate the existence of carbon, nitrogen, oxygen in all the three catalysts, along with sulphur in g-C_3_N_4_-(CH_2_)_3_-SO_3_H. The C 1s spectra in Fig. 4(b) deconvoluted in four components. The primary peak at 284.8 eV represents the adventitious carbon from impurities and/or sp^3^C from g-C_3_N_4_^28,49^. The second peak at 286.0 eV is associated with C-NH_2_ species^44^. Two additional peaks with higher binding energies, at 287.9 and 288.3 eV are attributed to N = C-N coordination and the N-C-O groups, respectively^50,51^. After functionalization, the binding energies of C1s of N = C-N and N-C-O species apparently shifts to higher binding energy value owing to the electronegative functional group attachment. In Fig. 4c, the deconvoluted N 1s spectra of pristine g-C_3_N_4_ reveal three peaks at 400.8, 399.6 and 398.1 eV corresponding to the C-NHx, N-(C)_3_, and C-N = C bonding structures, respectively^40^. Interestingly, the N 1s XPS spectra of functionalized g-C_3_N_4_-(CH_2_)_3_-SO_3_H and g-C_3_N_4_-CO-(CH_2_)_3_-CO_2_H catalysts exhibit similar peaks at higher binding energies of 402.0, 400.7, and 399.5 eV. As a result of –SO_3_H, -COOH functionalization, the peaks corresponding to s-triazine units in C-1s and N-1s XPS spectra of functionalized g-C_3_N_4_ nanosheets are shifted to the higher binding energies as compared to pristine g-C_3_N_4_^52^. Figure 4(d) shows the XPS spectrum of O 1s in functionalized g-C_3_N_4_-(CH_2_)_3_-SO_3_H. The oxygen atoms in S-OH and S = O were observed at 531.6 eV and 533.0 eV, respectively^53^. Figure 4(e) displays the S 2p XPS spectrum of functionalized g-C_3_N_4_-(CH_2_)_3_-SO_3_H. The deconvoluted peaks at 168.0 eV and 168.9 eV correspond to S 2p_3/2_ and S 2p_1/2_, respectively from the -SO_3_H group^44,54^.

**Fig. 5:**
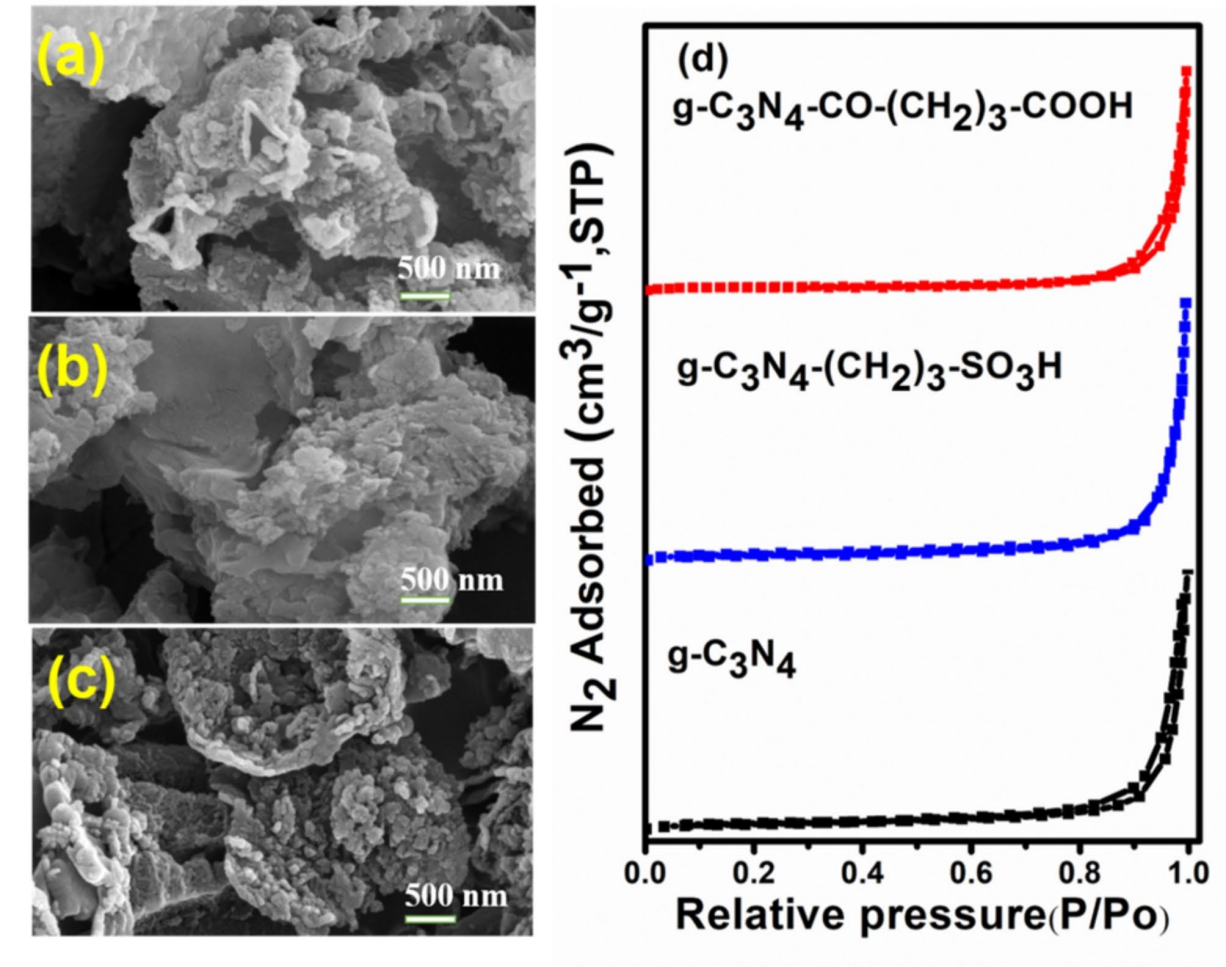
FE-SEM micrographs of (**a**) g-C_3_N_4_, (**b**) g-C_3_N_4_-(CH_2_)_3_-SO_3_H, (**c**) g-C_3_N_4_-CO-(CH_2_)_3_-CO_2_H and (**d**) N_2_ sorption isotherm of pristine and functionalized g-C_3_N_4_.

FE-SEM analyses presented in Fig. 5(a–c) elucidated the morphology of pristine g-C_3_N_4_ and functionalized g-C_3_N_4_ catalysts. g-C_3_N_4_ exhibited aggregated and crumbled sheet-like morphology, while g-C_3_N_4_-CO-(CH_2_)_3_-CO_2_H along with g-C_3_N_4_-(CH_2_)_3_-SO_3_H displayed a stacked sheet morphology. The BET analysis was carried out to measure the surface area of g-C_3_N_4_, functionalized g-C_3_N_4_-(CH_2_)_3_-SO_3_H, and g-C_3_N_4_-CO-(CH_2_)_3_-CO_2_H catalysts. The nitrogen adsorption and desorption isotherms of the three synthesized catalysts are shown in Fig. 5d. These isotherms exhibited a characteristic Type IV physisorption pattern and an H_3_ hysteresis loop. Based on the Brunauer-Deming-Teller classification, the g-C_3_N_4_ and functionalized-g-C_3_N_4_ catalysts have slit-shaped mesopores that resulted from the stacking of thin nanosheets^55^. The surface area of the g-C_3_N_4_ catalyst was 23.53 m^2^ g^− 1^, whereas g-C_3_N_4_-(CH_2_)_3_-SO_3_H and g-C_3_N_4_-CO-(CH_2_)_3_-CO_2_H exhibited a surface area of 14.26 and 31.19 m^2^ g^− 1^, respectively. The mean pore size for g-C_3_N_4_, functionalized g-C_3_N_4_-(CH_2_)_3_-SO_3_H, and g-C_3_N_4_-CO-(CH_2_)_3_-CO_2_H catalysts was found in the range of 35.00 to 42.00 nm, whereas the mean pore volume for each catalyst was in the range of 0.15 to 0.29 cm^3^ g^− 1^.

### Catalytic activity

**Fig. 6:**
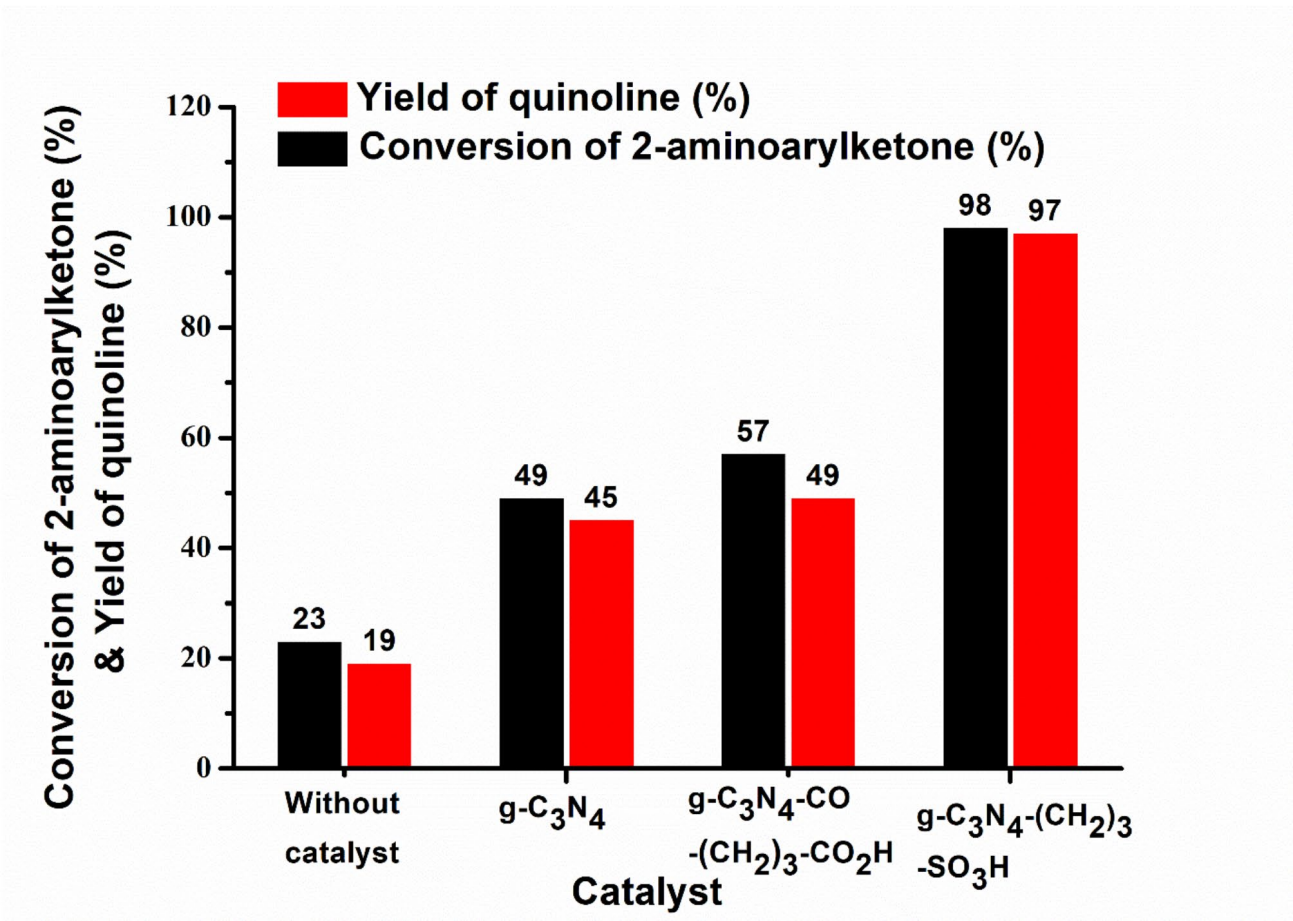
Conversion of 2-aminoarylketone (%) and yield of quinolines (%) over heterogeneous acid catalysts and without catalyst. Reaction condition: 2-aminoaryl ketone (1.00 mmol), acetyl acetone (1.2 mmol) and 10.0 wt % of catalyst loading without solvent at 100 °C for 4 h.

The thoroughly characterized catalysts were screened for synthesis of biologically and physiologically attractive quinoline derivatives from 2-aminoarylketone with acetyl acetone. Initially, catalysts were studied under consistent experimental conditions, specifically without any solvent at a temperature of 100 °C for 4 h of duration. Quantitative NMR analysis with an internal standard 1,3,5-trimethoxybenzene was carried out to determine the conversion and yield of the reaction. The catalytic results are shown in Fig. 6. The pristine g-C_3_N_4_ material facilitated the conversion of 49% of the 2-aminoarylketone, resulting in a 45% yield of the desired product, while the control experiment without any catalyst yielded only 19% of quinoline, with a 23% conversion of 2-aminoarylketone. Apparently, the catalyst played a crucial role in the Friedländer synthesis of quinoline, as demonstrated by the results. The rate of reaction over the pristine g-C_3_N_4_ was determined to be 1.19 × 10^− 6^ mol g^− 1^ s^− 1^. Further catalytic screenings were conducted using Bronsted acid functionalized g-C_3_N_4_ catalysts. It is apparent from Fig. 6 that g-C_3_N_4_-CO-(CH_2_)_3_-CO_2_H could has a marginally impact on enhancing the conversion rate of 2-aminoarylketone to 57% and increasing the yield of quinoline to 49%. Remarkably, g-C_3_N_4_-(CH_2_)_3_-SO_3_H demonstrated exceptional efficiency, achieving a 98% conversion rate of 2-aminoarylketone to quinoline, accompanied by a notably elevated yield of 97%. The calculated rate was 1.19 × 10^− 6^ and 2.77 × 10^− 5^ mol g^− 1^ s^− 1^ over g-C_3_N_4_-CO-(CH_2_)_3_-CO_2_H and g-C_3_N_4_-(CH_2_)_3_-SO_3_H, respectively. The atom economy of the conversion of 2-aminoarylketone to the corresponding quinoline over g-C_3_N_4_-(CH_2_)_3_-SO_3_H without considering the water was found to be 90.13%. The findings piqued our interest to compare the effectiveness of our material with the catalysts reported in the open literature. Table 1 provides a summary of the efficiency of acid-based heterogeneous catalysts in the Friedländer synthesis of quinoline. The investigations in the table demonstrate an operational reaction temperature of 100 °C or less, with or without solvents. Remarkably, g-C_3_N_4_-(CH_2_)_3_-SO_3_H demonstrated exceptional performance, surpassing the catalysts mentioned in previous studies.

**Table 1:** Performance evaluation of our catalyst in comparison to literature data.

Catalyst	Temp (°C)	Solvent	Time (h)	Yield (%)	Catalyst loading (mol%)	Rate of reaction (mol s^− 1^ g^− 1^)	References
NiO NPS	100	Solvent free	3.0	91	10	3.36 × 10^− 6^	^56^
Nafion NR50	200	Solvent free	1.0	88	20	5.48 × 10^− 6^	^57^
PMA·SiO2	78	Ethanol	1.0	88	10	1.09 × 10^− 5^	^23^
Silver Phosphotungstate	78	Ethanol	5.0	89	20	9.88 × 10^− 7^	^58^
Amberlyst 15	78	Ethanol	2.5	75	50	2.22 × 10^− 7^	^22^
Propyl sulfonic silica(PSS)	80	Solvent free	5.0	80	20	8.88 × 10^− 7^	^59^
Sulfamic acid	70	Solvent free	1.0	95	5	2.11 × 10^− 5^	^60^
g-C_3_N_4_-(CH_2_)_3_-SO_3_H	100	Solvent free	4.0	97	10	2.77 × 10^− 5^	Present work

**Fig. 7:**
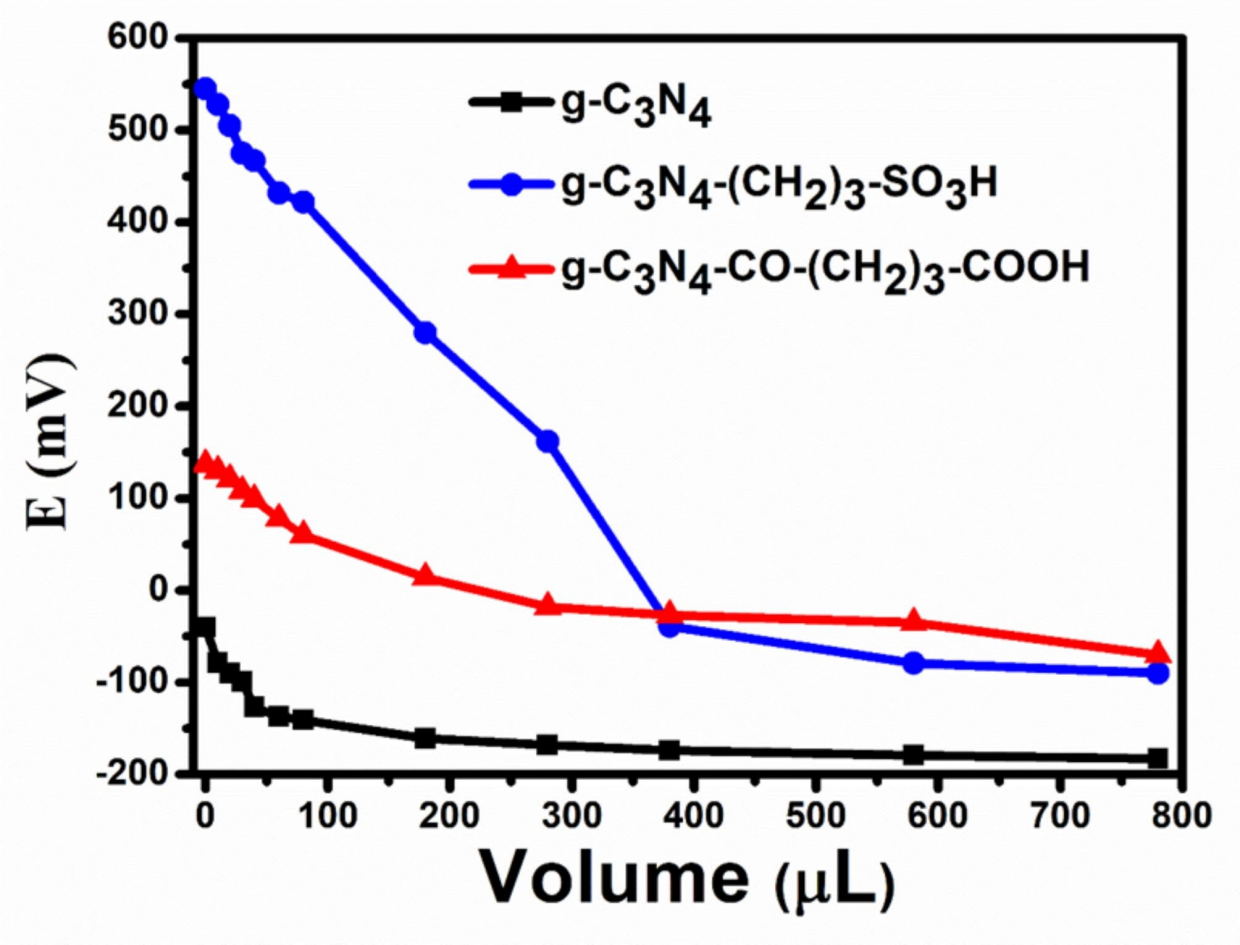
Potentiometric titration curves of the synthesized g-C_3_N_4_ and acid functionalized g-C_3_N_4_-CO-(CH_2_)_3_-CO_2_H and g-C_3_N_4_-(CH_2_)_3_-SO_3_H.

Apparently, the pivotal acid sites play a vital role in augmenting the Friedländer synthesis of quinoline, as also suggested by the results from Table 1. Therefore, evaluation of strength of the acid site of the catalysts is very important for corelating the conversion efficiency of 2-aminoarylketone in quinolines. The potentiometric titration technique was used to evaluate the acidity potency of the synthesized pristine and functionalized g-C_3_N_4_ catalysts. This method evaluates the acidity of the catalyst, making it possible to determine the total number of acidic sites in addition to the strength of each individual site. The initial electrode potential (E in mV) was employed for assessing the strength of acid sites (in M.eq/g solid catalyst), and Fig. 7 shows the titration curves for the synthesised catalysts. These acid sites are categorized into four groups: E > 100 mV (indicating very strong acid sites), 0 < E < 100 mV (representing moderate acid sites), -100 < E < 0 mV (characterizing weak acid sites), and E < -100 mV (signifying very weak acid sites)^61,62^. The results clearly demonstrated that g-C_3_N_4_-(CH_2_)_3_-SO_3_H exhibited an exceptionally high initial electrode potential of 549 mV, indicative of the presence of very strong Brønsted acid sites. On the contrary, the initial electrode potentials of g-C_3_N_4_-CO-(CH_2_)_3_-CO_2_H and pristine g-C_3_N_4_ were 143 mV and − 44 mV, respectively, indicating the presence of moderate and weak acid sites over the materials. The findings of the acid strength rationalize the effectiveness of converting 2-aminoarylketone and yield of quinoline over pristine g-C_3_N_4_ and acid functionalized g-C_3_N_4_-CO-(CH_2_)_3_-CO_2_H and g-C_3_N_4_-(CH_2_)_3_-SO_3_H.

**Fig. 8:**
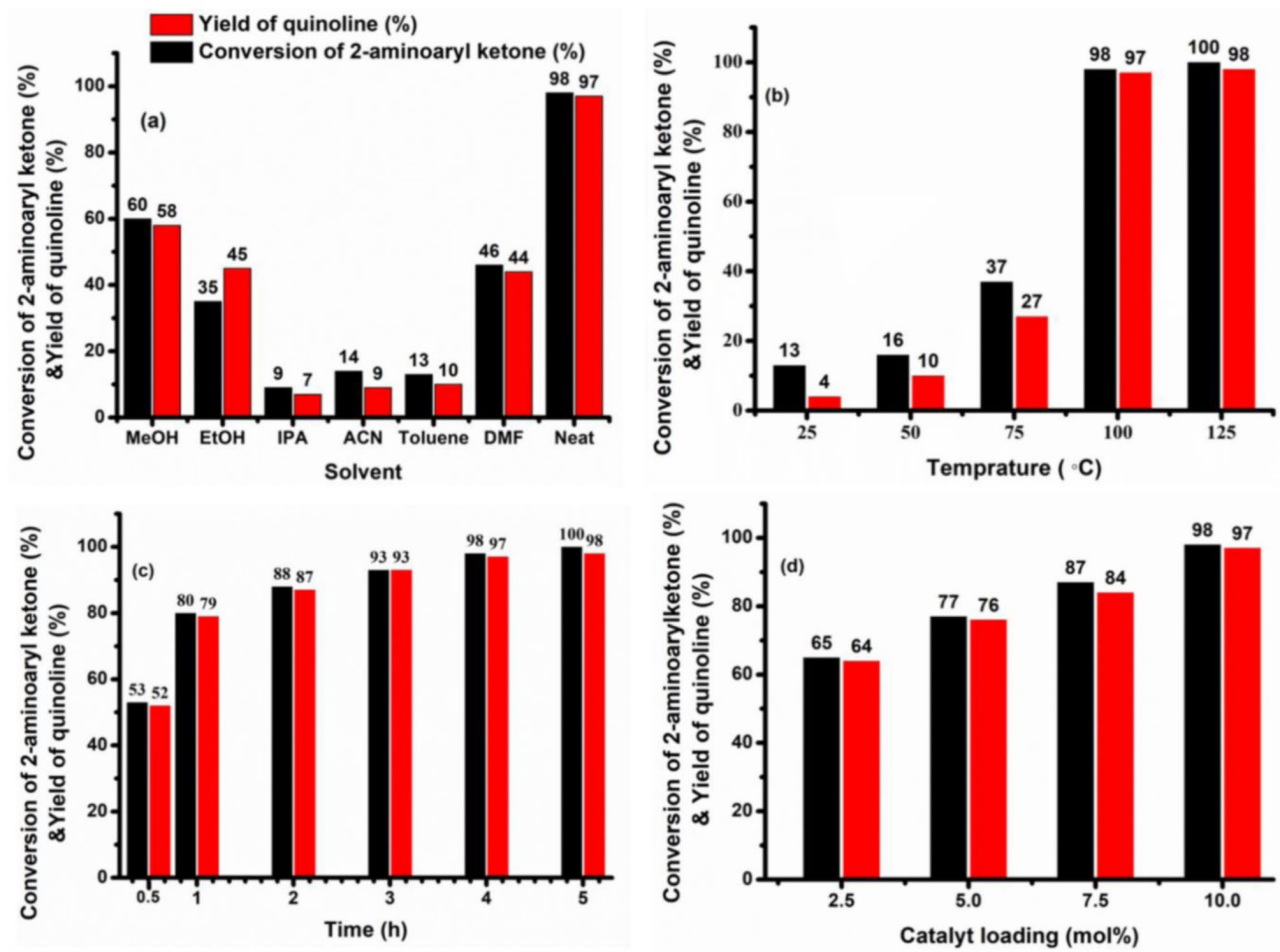
Optimization of reaction condition with g-C_3_N_4_-(CH_2_)_3_-SO_3_H: (a) variation of solvents, (b) temperature, (c) duration and (d) catalyst loading, other conditions 2-Aminoaryl ketone (1.0 mmol), acetyl acetone (1.2 mmol).

As g-C_3_N_4_-(CH_2_)_3_-SO_3_H turned out to be the best catalyst for synthesis of quinoline from 2-aminoarylketone, further the reaction conditions were optimized with this catalyst by changing the solvents, reaction temperature, duration, and catalyst loading. To optimise the solvent condition, the catalytic reactions were conducted with 10.0 wt% of g-C_3_N_4_-(CH_2_)_3_-SO_3_H catalyst loading for 4 h of duration. Commercially available solvents, such as ethanol (EtOH), Methanol (MeOH), Isopropanol (IPA), acetonitrile (ACN), toluene and N, N dimethyl formamide (DMF) were investigated. Figure 8(a) shows 2-aminoaryl ketone conversion and quinoline yield against different solvents. Among the solvents investigated, ethanol, methanol, and DMF demonstrated a yield of 40–60% for quinoline. In contrast, toluene and acetonitrile showed lower conversion rates for 2-aminoaryl ketones to quinoline derivative. The highest reactivity of g-C_3_N_4_-(CH_2_)_3_-SO_3_H was observed in the absence of a solvent with a product yield of 97% and 98% conversion of 2-amino aryl ketone. Therefore, the subsequent experiments were aimed at optimising the reaction conditions without any solvent. Figure 8(b) shows the percentage yield of quinoline without any solvent at different temperatures for 4 h of duration. The conversion of 2-aminoarylketone was initially minimal at room temperature. With a systematic increase of temperature by 25 °C for each successive experiment, the highest yield of quinoline was observed at 100 °C. Consequently, the reaction temperature was optimised at 100 °C thereafter. Further, the reaction duration was varied from 0.5 to 5.0 h under solvent-free conditions while keeping the reaction temperature constant at 100 °C over 10.0% g-C_3_N_4_-(CH_2_)_3_-SO_3_H. Figure 8(c) displays that a duration of 4 h is the optimal time for the Friedländer synthesis of quinoline. Finally, the optimisation of the g-C_3_N_4_-(CH_2_)_3_-SO_3_H catalyst loading was achieved by changing the weight% from 0 to 10.0%. The results shown in Fig. 8(d) indicate that there is minimal advancement of the reaction in the absence of catalysts under the given reaction conditions. A 10.0 wt% loading of the g-C_3_N_4_-(CH_2_)_3_-SO_3_H catalyst achieved the best percentage yield of quinoline.

**Fig. 9:**
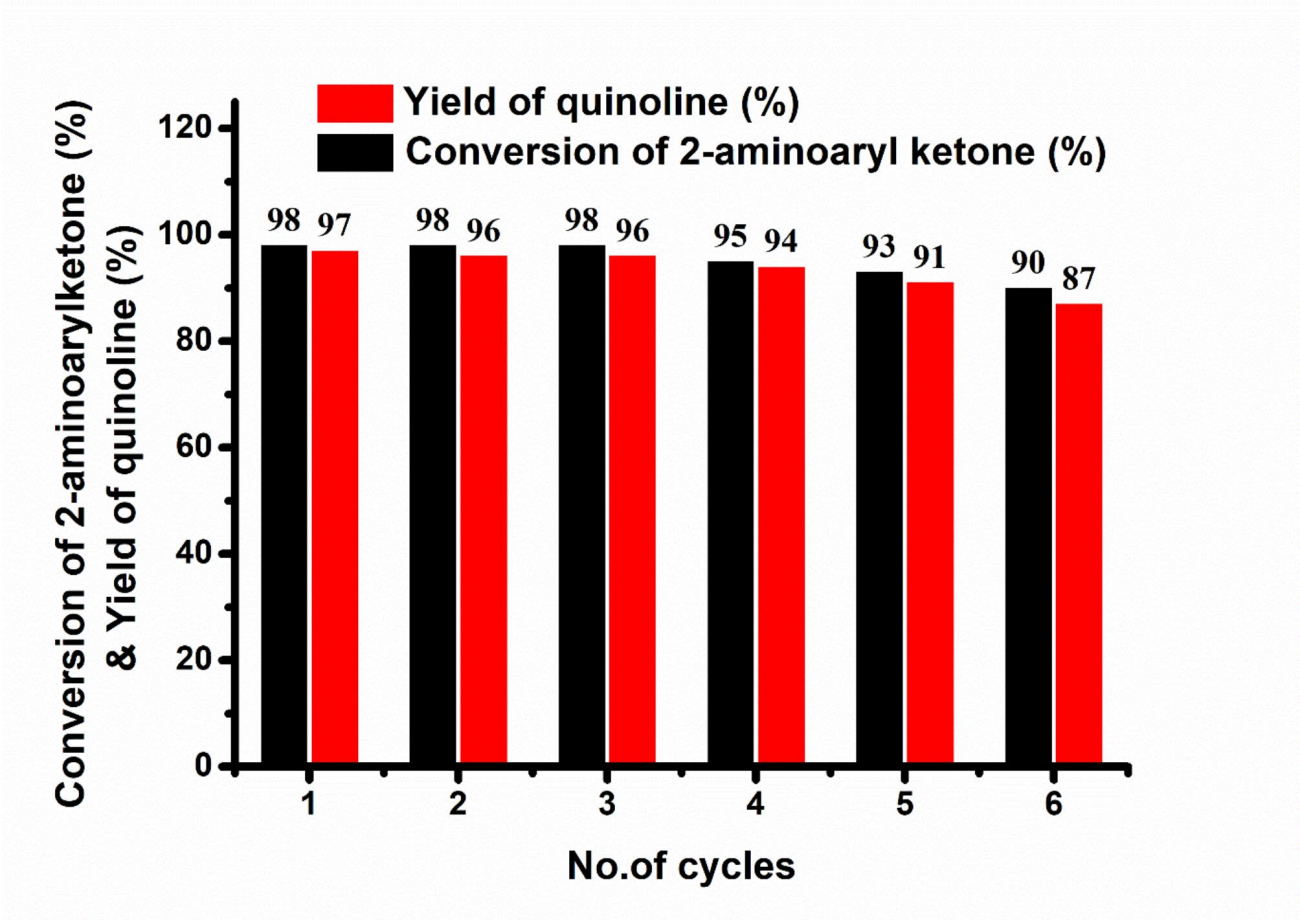
Recyclability of g-C_3_N_4_-(CH_2_)_3_-SO_3_H with optimized reaction conditions.

The optimisation experiments revealed that a 10 wt% loading of g-C_3_N_4_-(CH_2_)_3_-SO_3_H catalyst at 100 °C could maximise quinoline yield under solvent-free conditions in 4 h of reaction time. When considering the possible practical applications, the main benefit of using heterogeneous acid catalysts is their reusability and stability, in addition to their greater catalytic activity. Therefore, the recyclability of the catalysts g-C_3_N_4_-(CH_2_)_3_-SO_3_H under the optimised reaction conditions for up to 6 cycles was investigated. After each catalytic cycle, the catalyst was retrieved by centrifugation, using dichloroethane, and subsequently was dried under normal atmospheric conditions. Figure 9 demonstrates the recyclability of the exhausted catalyst, indicating that the catalytic activity remains intact for up to 6 consecutive cycles. The yield of quinoline was marginally dropped from 97 to 87%. After six cycles, the XRD, XPS, FE-SEM, FTIR and CHNS tests revealed that the catalyst retained its structure, morphology, and surface chemical compositions (Table S1, and Figure S1). The potentiometric titration experiment exhibited a marginal loss of acid strength in the exhausted catalyst. The synthesized g-C_3_N_4_-(CH_2_)_3_-SO_3_H with Brønsted acid functionality demonstrated remarkable stability throughout numerous catalytic cycles.

Further experiments were carried out to evaluate the scope of the substrate of the reaction with respect to various α-methylene carbonyl and 2-aminoaryl ketone compounds over the best catalysts g-C_3_N_4_-(CH_2_)_3_-SO_3_H at optimized reaction conditions. Different substituted α-methylene carbonyl derivatives were reacted with 2-aminoaryl ketone substituted derivatives, and the NMR and MS data were utilised to characterise all the obtained products (Figures S2-S25), with results summarized in Table 2. When compared with unsubstituted 2-aminoaryl ketone, the yield of quinoline was slightly lower in 2-aminoaryl ketone that has a halogen or nitro group in para position to the amino group on the aromatic ring. When 2-aminobenzaldehyde or 2-aminoacetophenone were reacted instead of 2-aminobenzophenone in the reaction with acetylacetone, the resulting quinoline derivatives were obtained with yields of 77% and 86%, respectively. The corresponding quinoline derivative yield decreased from 97 to 86% when 2’,5-dichlorobenzophenone was combined with ethyl acetoacetate instead of acetyl acetone. Similarly, when ethyl acetoacetate was used instead of acetyl acetone in the reaction with 2-aminoacetophenone, a decrease in yield of the corresponding quinoline derivative from 86 to 80% was observed. When reacting with 2’,5-dichlorobenzophenone, six-membered cyclohexanone and 1,3-dicyclohexanone showed a comparable percentage yield of the desired product (~ 96%) among the cyclic ketones. The high yield observed may be attributed to the stabilization of nucleophiles by six-membered ring ketones. This pattern was consistent in the synthesis of quinoline derivatives when 2-aminoacetophenone reacted with six-membered cyclic ketones as well. However, the presence of ring strain in the five-membered cyclopentanone led to a lower yield of 85% compared to the equivalent quinoline derivative. Interestingly, phenyl substituted α-methylene carbonyl substrates exhibited lower yield of quinoline due to the electro withdrawing nature of the aromatic ring. Our analysis of the likely reaction mechanism is aided by this substrate’s scope investigations.

**Table 2:** Substrate scope of quinoline derivatives over g-C_3_N_4_-(CH_2_)_3_-SO_3_H catalyst under optimized reaction condition of 5 mol % of catalyst under solvent less in 4 h reaction durations.


Entry	2-aminoaryl ketones Substrate	α-methylene carbonyl substrate	Quinolines substrate	Isolated Yield (%)
1				77
2				86
3				93
4				89
5				97
6				86
7				95
8				96
9				85
10				80
11				94
12				80
13				91
14				81
15				72
16				64

**Fig. 10:**
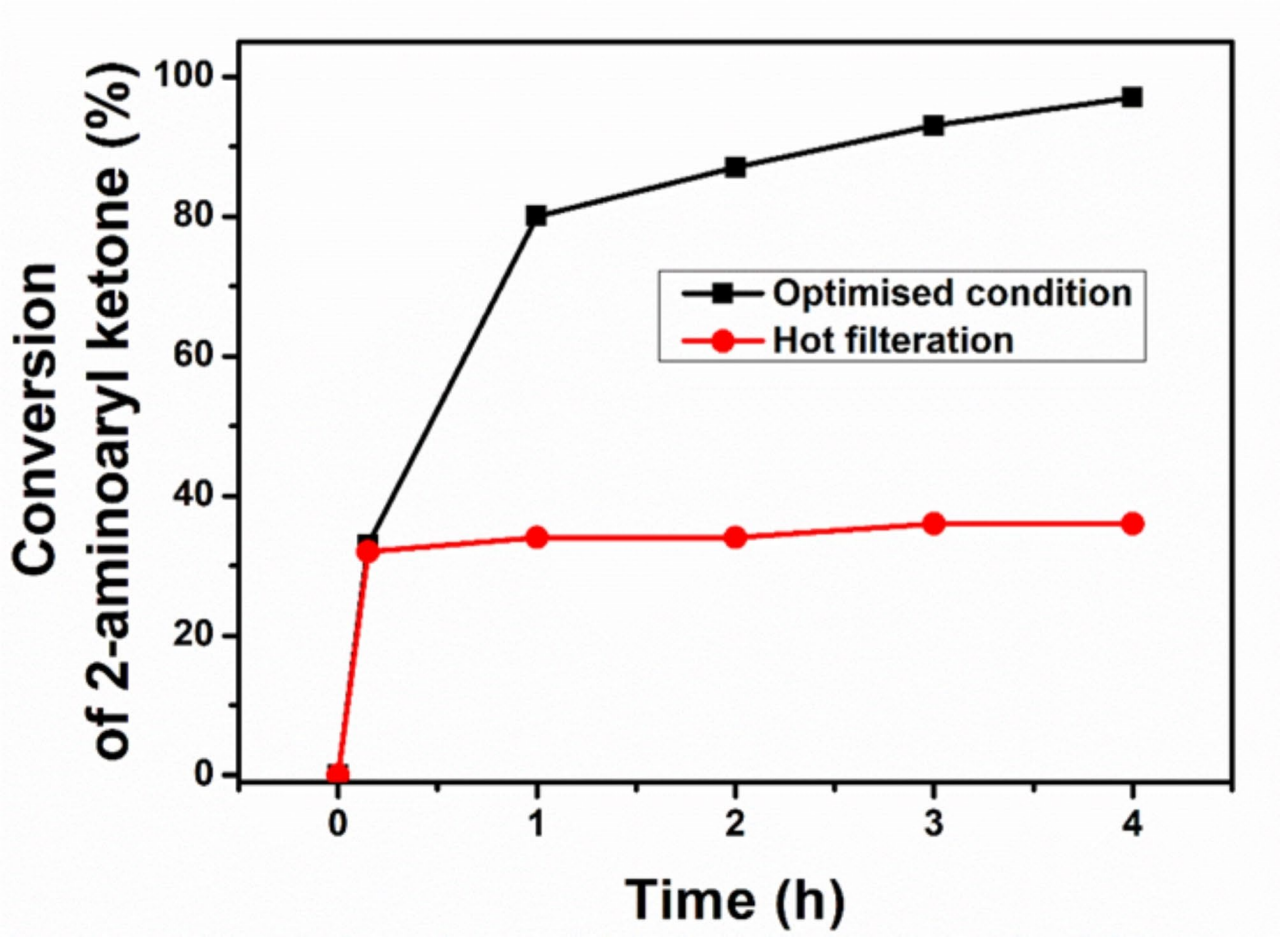
Hot filtration experiment of conversion of 2-aminoaryl ketone with and without g-C_3_N_4_-CO-(CH_2_)_3_-SO_3_H.

Numerous experimental and theoretical studies have investigated the Friedländer reaction mechanism over homogeneous as well as metal containing heterogeneous acid catalysts^63,64^. We probed the Friedländer reaction’s working mechanism over the synthesized metal-free heterogeneous catalyst, g-C_3_N_4_-(CH_2_)_3_-SO_3_H with the help of the surface characterization data of the catalyst, reaction intermediates and substrate scope studies. The hot filtration experiments were carried out to establish the importance of the Brønsted acid sites of the g-C_3_N_4_-(CH_2_)_3_-SO_3_H. After 15 min of the commencement of the reaction in the optimized condition, the catalyst was removed from the reaction mixture by filtration using a hot frit, resulting in only 33% conversion of 2-aminoaryl ketone. The filtrate was then monitored for an additional 4 h to evaluate reaction progress using quantitative^1^HNMR. The results (Fig. 10) demonstrate that after the removal of the active catalyst, the reaction did not proceed, establishing the importance of the Brønsted acid sites of the catalyst g-C_3_N_4_-CO-(CH_2_)_3_-SO_3_H.

**Fig. 11:**
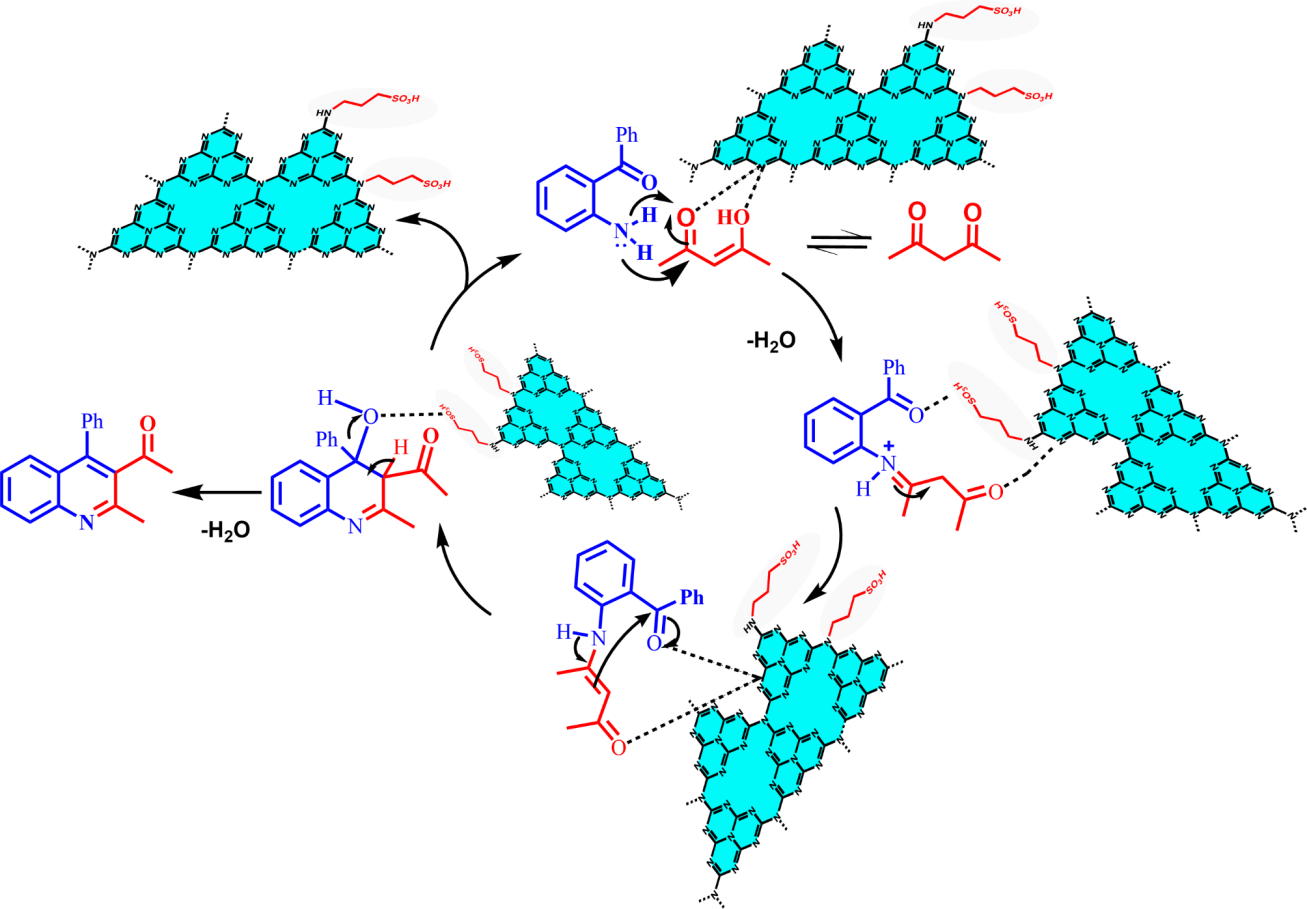
Reaction mechanism pathway of quinoline derivatives from 2-aminoaryl ketone in presence of α-methylene carbonyl derivatives over g-C_3_N_4_-(CH_2_)_3_-SO_3_H.

In order to obtain the stable reaction intermediates, the Friedländer reaction was conducted under optimized experimental conditions over g-C_3_N_4_-(CH_2_)_3_-SO_3_H for four hours at room temperature, and the intermediates were examined using LCMS and HPLC methods. Based on the experimental observation, a probable mechanism is shown in Fig. 11. At the beginning, the reactant acetyl acetone may adsorb on the g-C_3_N_4_-(CH_2_)_3_-SO_3_H catalyst surface through formation of hydrogen bond with C-NH_x_ of g-C_3_N_4_. The terminal tri-s-triazine unit of g-C_3_N_4_ comprises different types of nitrogen such as (i) pyridinic nitrogen, (ii) graphitic nitrogen, (iii) oxidic nitrogen, (iv) tertiary and Lewis based nitrogen, and (v) pyrrolic nitrogen as presented schematically in the figure below. The various N-species in g-C_3_N_4_ are demonstrated to be very effective for catalytic reactions as they impart H-bonding, tunable basicity, Lewis acidity. It should be noted that the existence of C-NH_x_ species was found to be highest over g-C_3_N_4_-(CH_2_)_3_-SO_3_H from XPS studies. The following step is condensation of amine group in 2-aminoaryl ketone derivative and acetyl acetone with the help of protonation from the Brønsted sites of the catalyst g-C_3_N_4_-(CH_2_)_3_-SO_3_H, leading to imine intermediate, which can tautomerize to enamines. The LCMS and HPLC data identified the formation of imine intermediate (Figure S26). The intramolecular aldol reaction in the next step produces the cyclized intermediate, which eventually loses water and yields the anticipated quinoline derivative. The reaction scheme prominently shows the importance of the Brønsted acid sites present in g-C_3_N_4_-(CH_2_)_3_-SO_3_H. The -SO_3_H group in g-C_3_N_4_-(CH_2_)_3_-SO_3_H played a pivotal role in both the imine production and the cyclization processes.

## Conclusion

g-C_3_N_4_ was successfully synthesized from melamine, followed by reactions with glutaric anhydride and 1,3-propanesultone, resulting in crystalline g-C_3_N_4_-CO-(CH_2_)_3_-CO_2_H and g-C_3_N_4_-CO-(CH_2_)_3_-SO_3_H, as confirmed by powder XRD and FTIR studies. Surface functionalization of g-C_3_N_4_ was further supported by detailed XPS analysis. Additional studies revealed no alteration in surface area, porosity after surface functionalization. The Friedländer synthesis of quinoline over pristine g-C_3_N_4_ showed a rate of 1.19 × 10^− 6^ mol g^− 1^ s^− 1^, while an order of magnitude higher rate of 2.77 × 10^− 5^ mol g^− 1^ s^− 1^ was observed over the g-C_3_N_4_-CO-(CH_2_)_3_-SO_3_H catalyst. This rate surpassed catalysts mentioned in previous studies, with notable recyclability. The high rate of Friedländer synthesis over g-C_3_N_4_-CO-(CH_2_)_3_-SO_3_H was attributed to its high surface acidity, as determined by potentiometric titration. Optimization experiments revealed that a 10 wt% loading of g-C_3_N_4_-CO-(CH_2_)_3_-SO_3_H catalyst at 100 °C maximized quinoline yield under solvent-free conditions within 4 h of reaction time. The Friedländer reaction mechanism over the synthesized metal-free heterogeneous catalyst, g-C_3_N_4_-CO-(CH_2_)_3_-SO_3_H, was probed using surface characterization data, reaction intermediates, and substrate scope studies. This study represents a unique and comprehensive exploration of metal-free heterogeneous catalysts for the Friedländer reaction.

### Supporting information summary

Supporting Information includes CHNS analysis^1^, H -NMR and^13^C- NMR, HPLC, LCMS, and the powder XRD, XPS and FE-SEM.

## Electronic supplementary material

Below is the link to the electronic supplementary material.

Supplementary Material 1

## Data Availability

All data generated or analysed during this study are included in this published article [and its supplementary information files].
