# Supplementary material for: Promising metal-free green heterogeneous catalyst for quinoline synthesis using Brønsted acid functionalized g-C3N4
Source: Sci Rep. 2024 Oct 10;14:23686. doi: 10.1038/s41598-024-72980-1 (PMC11467437; doi:10.1038/s41598-024-72980-1)
Supplement: Supplementary file 1 — Supplementary Material 1 [file 41598_2024_72980_MOESM1_ESM.docx]

**Promising Metal-Free Green Heterogeneous Catalyst for Quinoline Synthesis Using Brønsted Acid Functionalized g-C_3_N_4_**

**Bandarupalli Krishna ^[a,b]^, Sounak Roy*^[a,c]^**

*^a^ Department of Chemistry, Birla Institute of Technology and Science Pilani Hyderabad Campus, Hyderabad -500078, India*

*^b^Adama India Pvt. Ltd, Genome Valley Hyderabad-500078, India*

*^c^ Materials Centre for Sustainable Energy & Environment, Birla Institute of Technology and Science Pilani Hyderabad Campus, Hyderabad – 500078, India*

*Email: sounak.roy@hyderabad.bits-pilani.ac.in

| **Contents** |  | **Pages** |
| --- | --- | --- |
|  |  |  |
| Index |  | 1 |
| CHNS analysis of pristine and its functionalized g-C_3_N_4._ |  | 2 |
| Recovery catalysts,(a) XPS survey spectrum, (b) core level spectrum of S 2*p*, (c) XRD pattern and (d) FE-SEM micrographs of exhausted catalyst. |  | 3 |
| ^1^HNMR and ^13^CNMR 1-(2-methylquinolin-3-yl)ethan-1-one. |  | 4 |
| Mass spectrum of 1-(2-methylquinolin-3-yl)ethan-1-one & ^1^HNMR 1-(2,4-dimethylquinolin-3-yl)ethan-1-one. |  | 5 |
| ^13^CNMR and Mass spectrum 1-(2,4-dimethylquinolin-3-yl)ethan-1-one |  | 6 |
| ^1^HNMR and ^13^CNMR 1-(2-methyl-4-phenylquinolin-3-yl)ethan-1-one |  | 7 |
| Mass spectrum of 1-(2-methyl-4-phenylquinolin-3-yl)ethan-1-one and ^1^HNMR of 1-(2-methyl-6-nitro-4-phenylquinolin-3-yl)ethan-1-one |  | 8 |
| ^13^CNMR and Mass spectrum of 1-(2-methyl-6-nitro-4-phenylquinolin-3-yl)ethan-1-one. |  | 9 |
| ^1^HNMR and ^13^CNMR 1-(6-chloro-4-(2-chlorophenyl)-2-methylquinolin-3-yl)ethan-1-one |  | 10 |
| Mass spectrum of 1-(6-chloro-4-(2-chlorophenyl)-2-methylquinolin-3-yl)ethan-1-one &^1^HNMR Ethyl 6-chloro-4-(2-chlorophenyl)-2-methylquinoline-3-carboxylate . |  | 11 |
| ^13^CNMR and Mass spectrum of Ethyl 6-chloro-4-(2-chlorophenyl)-2-methylquinoline -3- carboxylate |  | 12 |
| ^1^HNMR and ^13^CNMR 7-chloro-9-(2-chlorophenyl)-1,2,3,4-tetrahydroacridine . |  | 13 |
| Mass spectrum 7-chloro-9-(2-chlorophenyl)-1,2,3,4-tetrahydroacridine &1HNMR of 7-chloro-9-(2-chlorophenyl)-3,4-dihydroacridin-1(2H)-one. |  | 14 |
| ^13^C NMR and Mass spectrum of 7-chloro-9-(2-chlorophenyl)-3,4-dihydroacridin-1(2H) -one. |  | 15 |
| ^1^HNMR and ^13^CNMR 7-chloro-9-(2-chlorophenyl)-2,3-dihydro-1H-cyclopenta [b]quinoline |  | 16 |
| Massspectrum7-chloro-9-(2-chlorophenyl)-2,3-dihydro-1H-cyclopenta [b]quinoline and ^1^HNMR Ethyl 2,4-dimethylquinoline-3-carboxylate |  | 17 |
| ^13^CNMR and mass spectrum Ethyl 2,4-dimethylquinoline-3-carboxylate. |  | 18 |
| ^1^HNMR and ^13^ CNMR 9-methyl-1,2,3,4-tetrahydroacridine. |  | 19 |
| Mass spectrum 9-methyl-1,2,3,4-tetrahydroacridine and ^1^HNMR 9-methyl-2,3-dihydro-1H-cyclopenta[b]quinoline |  | 20 |
| ^13^CNMR and mass spectrum of 9-methyl-2,3-dihydro-1H-cyclopenta[b]quinoline |  | 21 |
| Mass spectrum 9-methyl-2,3-dihydro-1H-cyclopenta[b]quinoline & ^1^HNMR 9-methyl-3,4-dihydroacridin-1(2H)-one |  | 22 |
| Mass spectrum 9-methyl-3,4-dihydroacridin-1(2H)-one &^1^HNMR of 6-chloro-4-(2-chlorophenyl)-2-phenylquinoline. |  | 23 |
| ^13^CNMR & Mass spectrum of 6-chloro-4-(2-chlorophenyl)-2-phenylquinoline. |  | 24 |
| ^1^HNMR and ^13^ CNMR of 1-(6-chloro-4-(2-chlorophenyl)-2-phenylquinolin-3-yl)ethan-1-one. |  | 25 |
| Mass spectrum of 1-(6-chloro-4-(2-chlorophenyl)-2-phenylquinolin-3-yl)ethan-1-one &^1^HNMR of (6-chloro-4-(2-chlorophenyl)-2-phenylquinolin-3-yl)(phenyl)methanone |  | 26 |
| ^13^CNMR and mass spectrum of (6-chloro-4-(2-chlorophenyl)-2-phenylquinolin-3-yl)(phenyl)methanone. |  | 27 |
| (a) HPLC chromatogram, (b) LCMS of 4-((4-chloro-2-(2-chlorobenzoyl) phenyl) imino) pentan-2-one.  ^1^H NMR,^13^C NMR and Mass results of products |  | 28 |

**Table S1**: CHNS analysis of pristine and its functionalized g-C_3_N_4_ .

| **Catalyst** | **Atomic (%) by CHNS** | | | |
| --- | --- | --- | --- | --- |
|  | **C** | **H** | **N** | **S** |
| g-C_3_N_4_ | 34.00 | 2.44 | 38.89 | 0.00 |
| g-C_3_N_4_-(CH_2_)_3_-SO_3_H | 32.20 | 2.27 | 45.09 | 2.07 |
| g-C_3_N_4_-CO-(CH_2_)_3_-CO_2_H | 42.00 | 7.65 | 50.36 | 0.00 |
| Exhausted  g-C_3_N_4_-(CH_2_)_3_-SO_3_H | 33.10 | 2.29 | 46.91 | 0.68 |


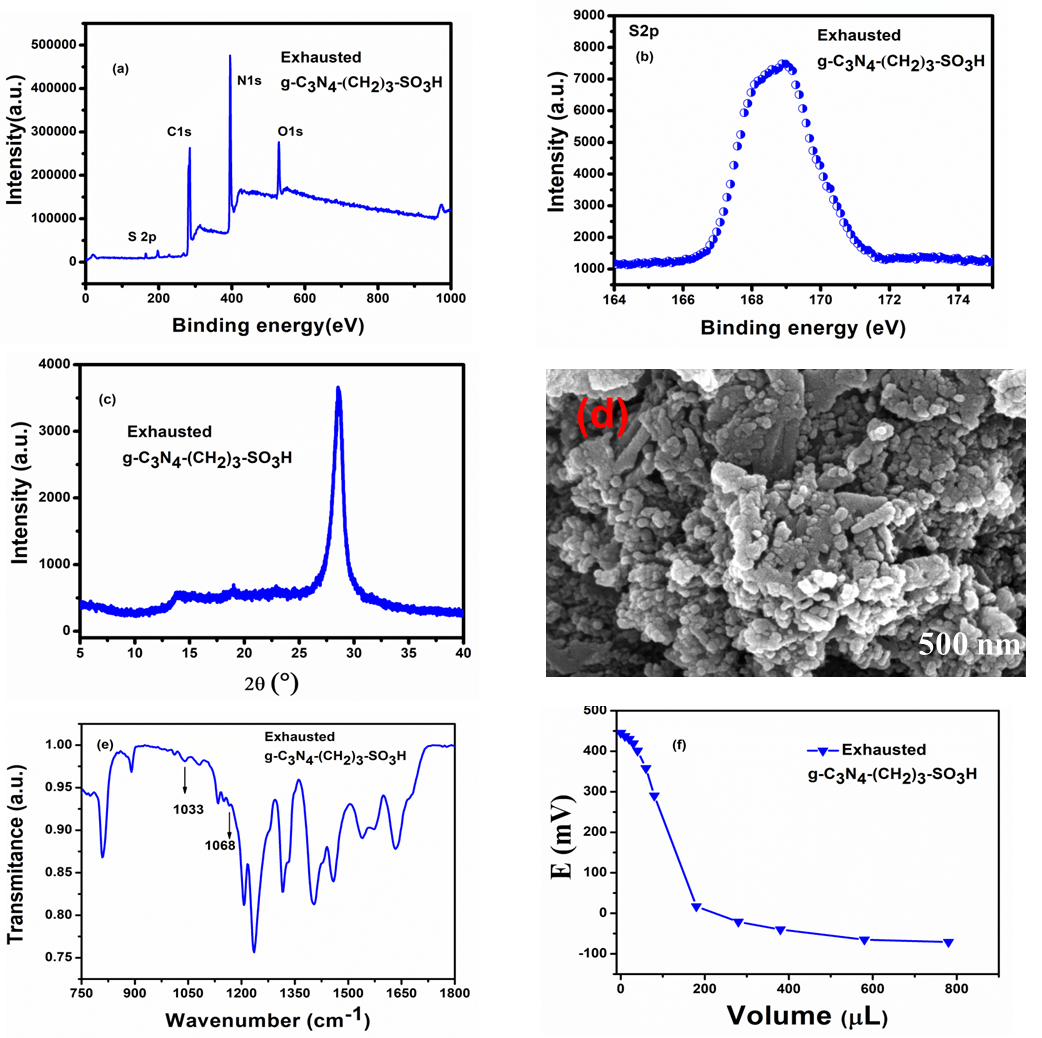


**Figure S1.** (a) XPS survey spectrum, (b) core level spectrum of S 2p, (c) XRD pattern, (d) FE-SEM micrograph, (e) FTIR spectrum, and (f) potentiometric titration curve of exhausted g-C_3_N_4_-(CH_2_)_3_-SO_3_H.

**
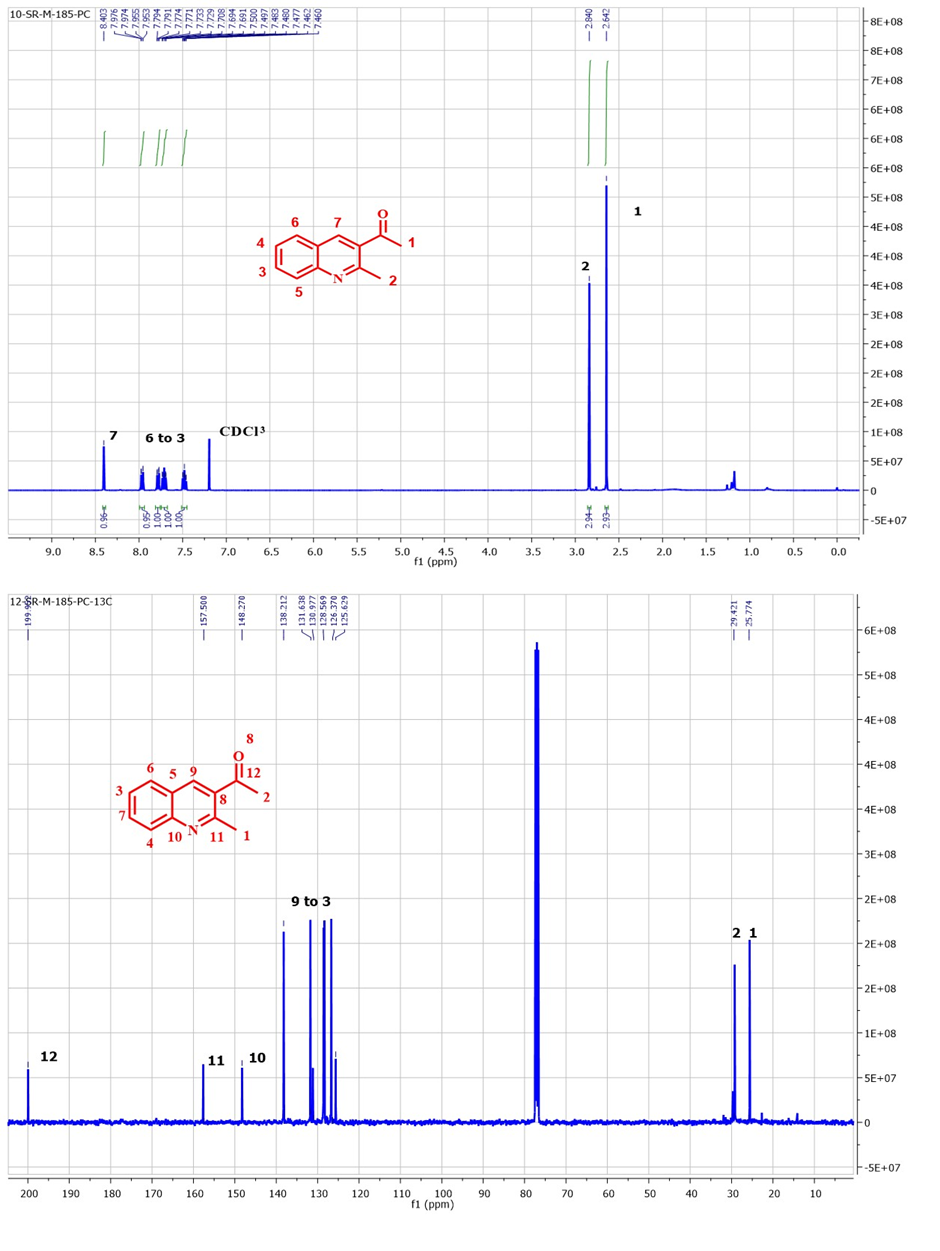
**

**Figure S2.** ^1^HNMR and ^13^CNMR 1-(2-methylquinolin-3-yl)ethan-1-one.


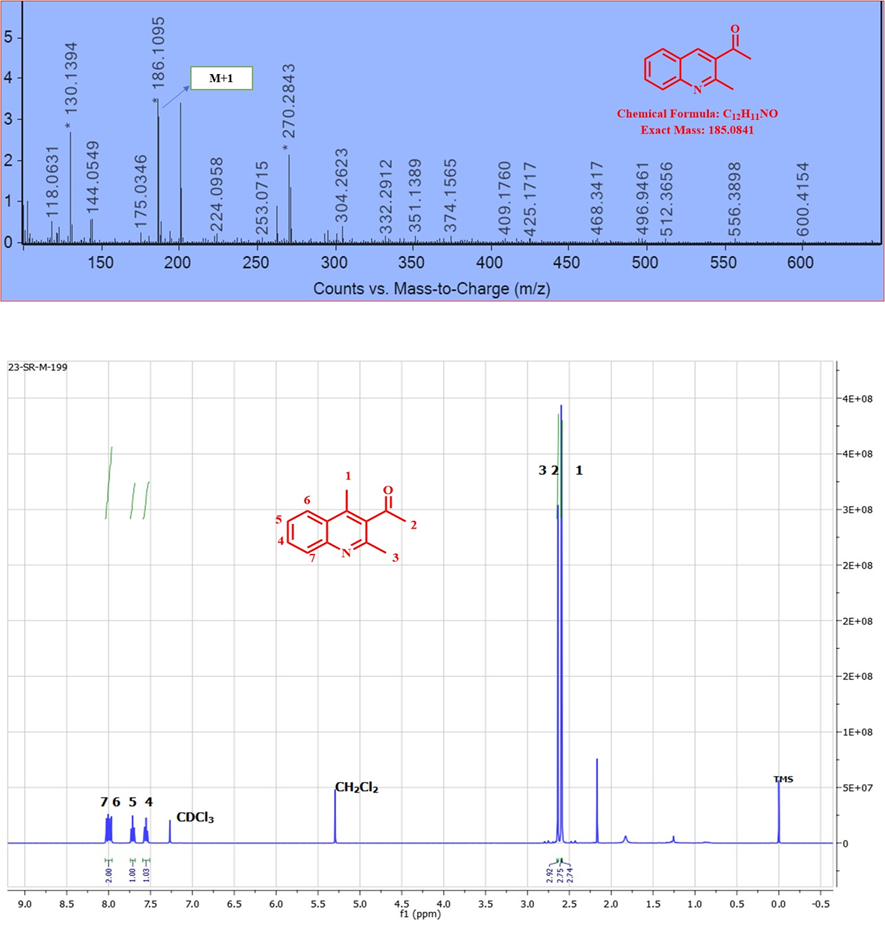


**Figure S3.** Mass spectrum of 1-(2-methylquinolin-3-yl)ethan-1-one & ^1^HNMR 1-(2,4-dimethylquinolin-3-yl)ethan-1-one.

**
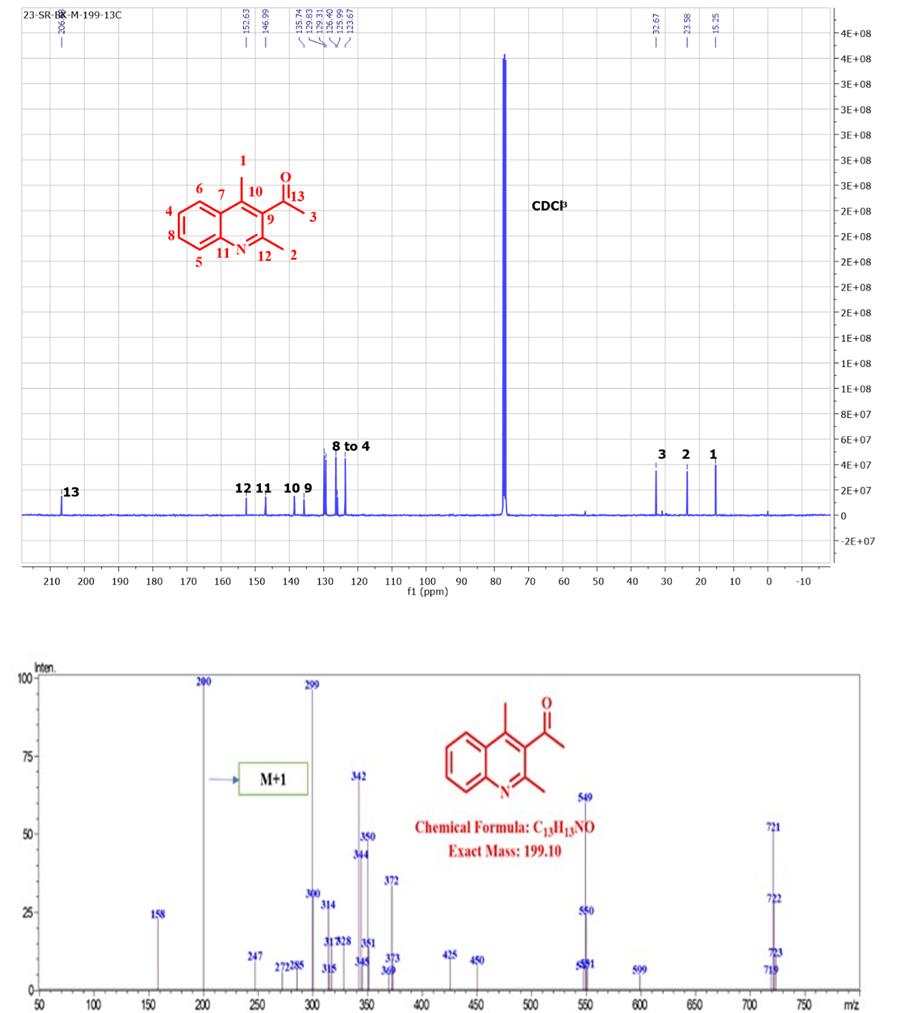
**

**Figure S4.** ^13^CNMR and Mass spectrum 1-(2,4-dimethylquinolin-3-yl)ethan-1-one.


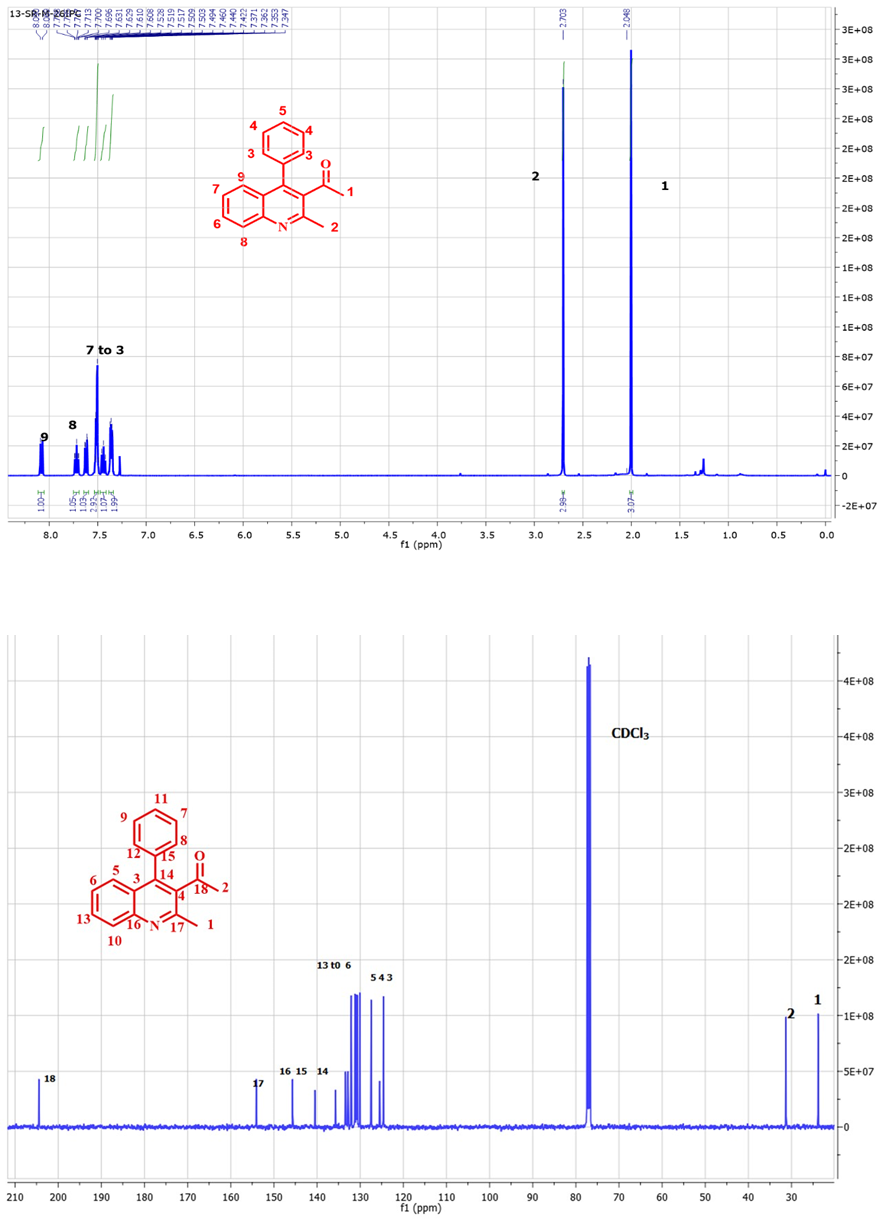


**Figure S5.** ^1^HNMR and ^13^CNMR of 1-(2-methyl-4-phenylquinolin-3-yl)ethan-1-one


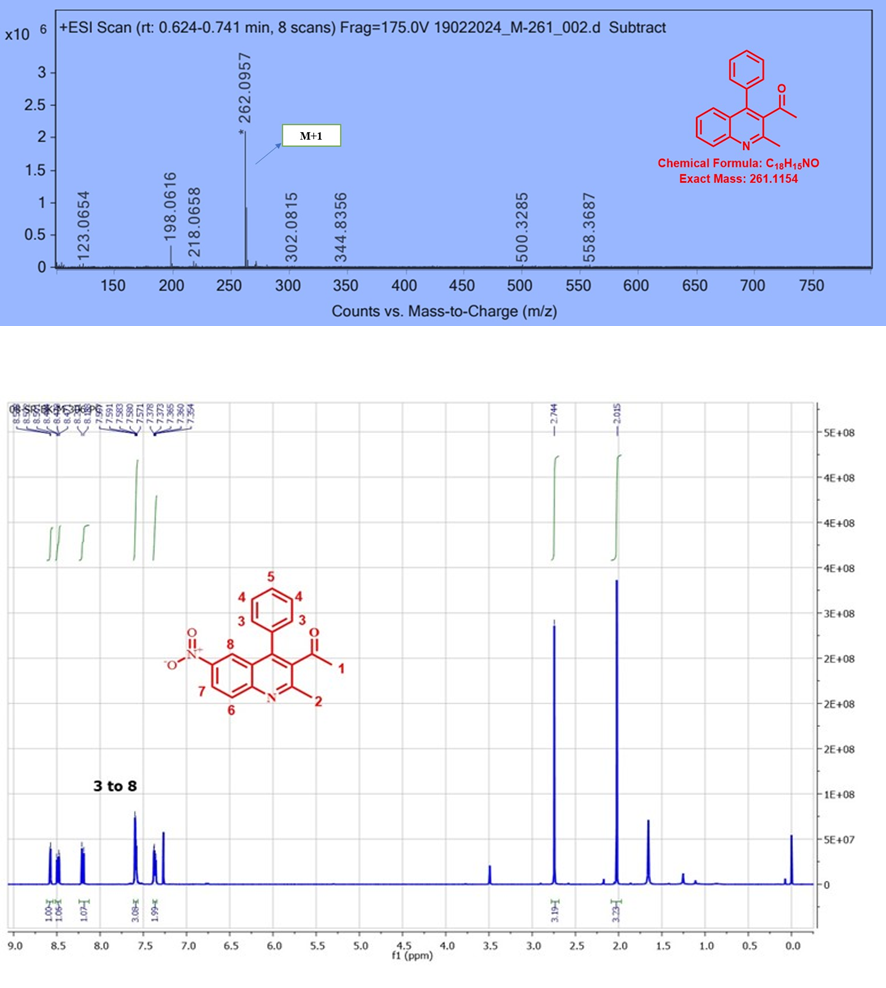


**Figure S6.** Mass spectrum of 1-(2-methyl-4-phenylquinolin-3-yl)ethan-1-one and ^1^HNMR of 1-(2-methyl-6-nitro-4-phenylquinolin-3-yl)ethan-1-one

**
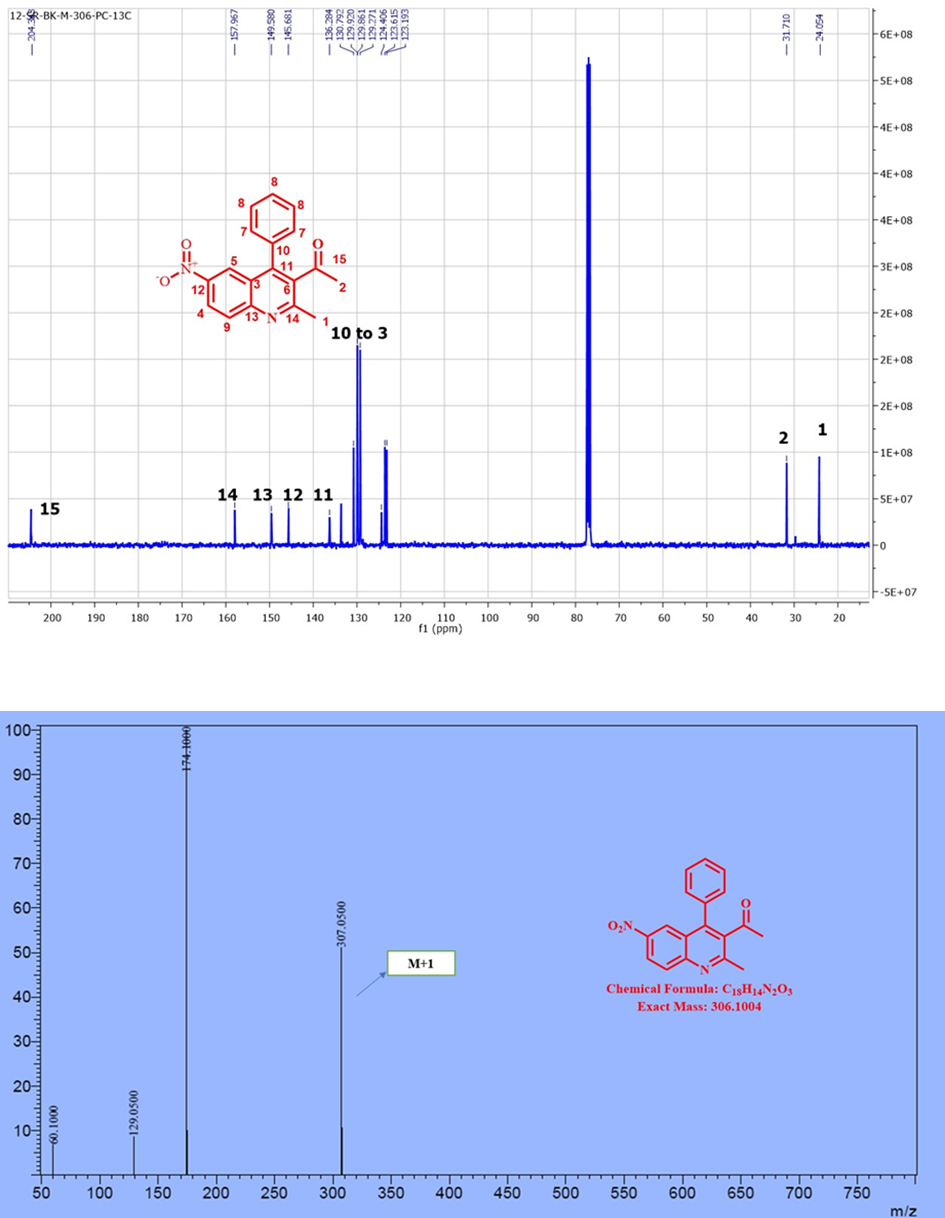
**

**Figure S7.** ^13^CNMR and Mass spectrum of 1-(2-methyl-6-nitro-4-phenylquinolin-3-yl)ethan-1-one.

**
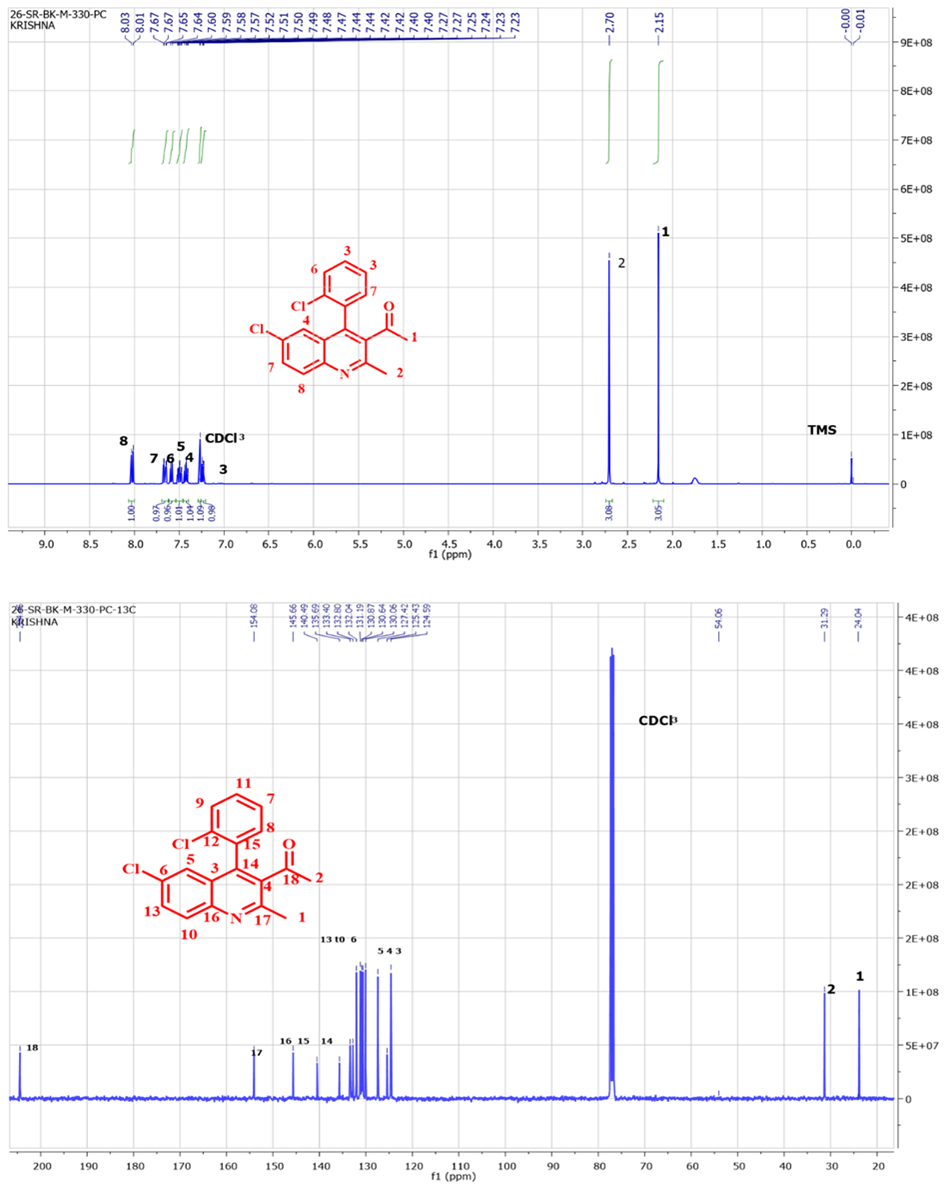
**

**Figure S8.** ^1^HNMR and ^13^CNMR 1-(6-chloro-4-(2-chlorophenyl)-2-methylquinolin-3-yl)ethan-1-one

**
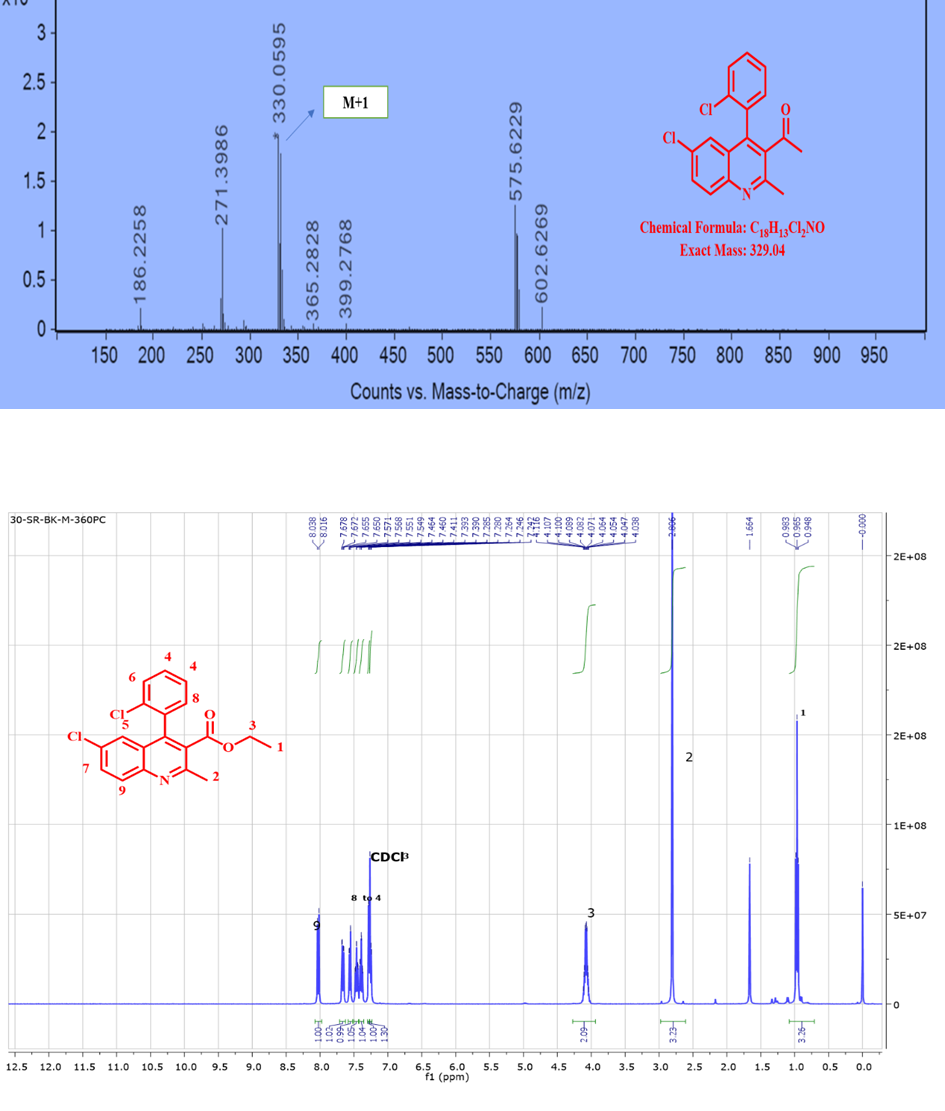
**

**Figure S** **9.** Mass spectrum of 1-(6-chloro-4-(2-chlorophenyl)-2-methylquinolin-3-yl)ethan-1-one &^1^HNMR Ethyl 6-chloro-4-(2-chlorophenyl)-2-methylquinoline-3-carboxylate


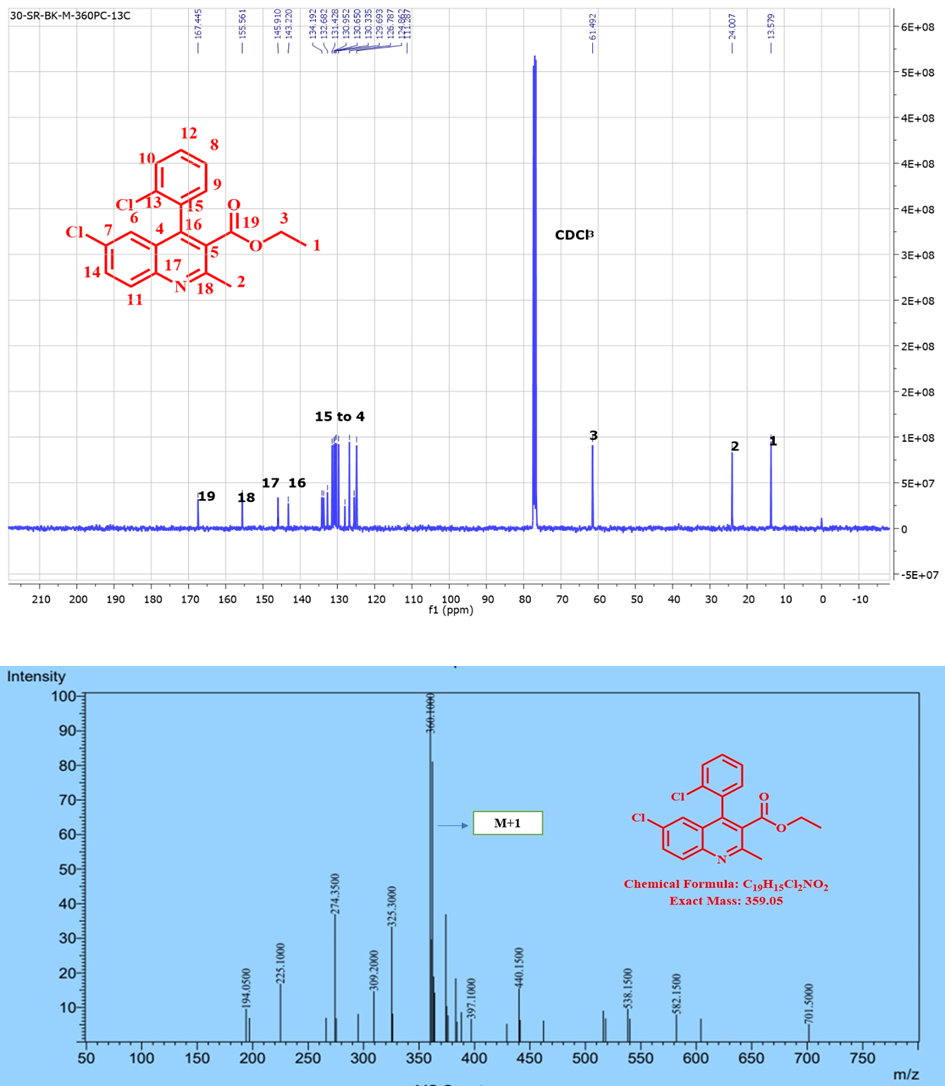


**Figure S10.** ^13^CNMR and Mass spectrum of Ethyl 6-chloro-4-(2-chlorophenyl)-2-methylquinoline-3-carboxylate

**M+1**


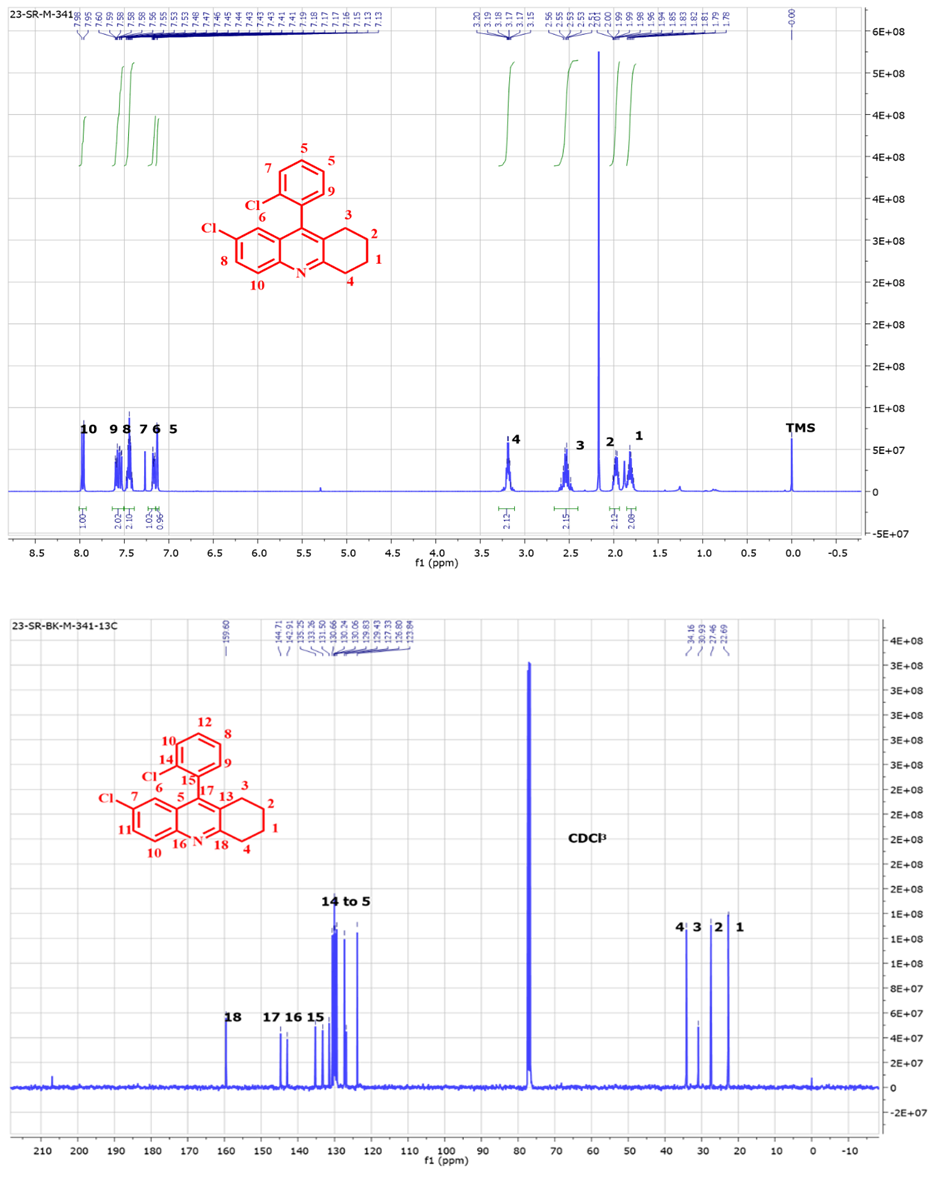


**Figure S11.** ^1^HNMR and ^13^CNMR 7-chloro-9-(2-chlorophenyl)-1,2,3,4-tetrahydroacridine


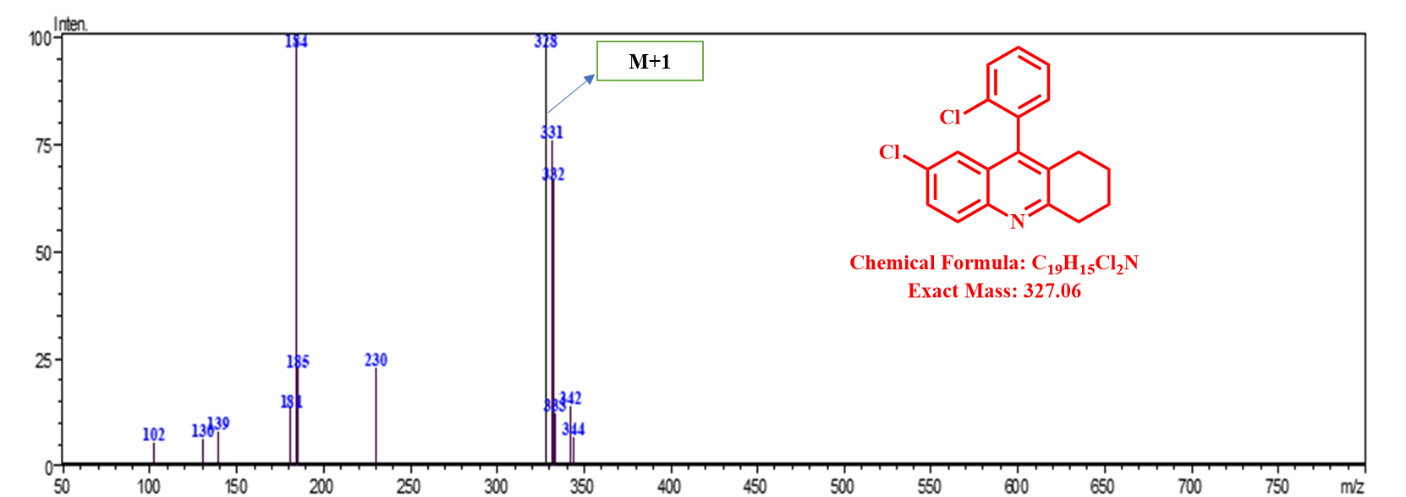

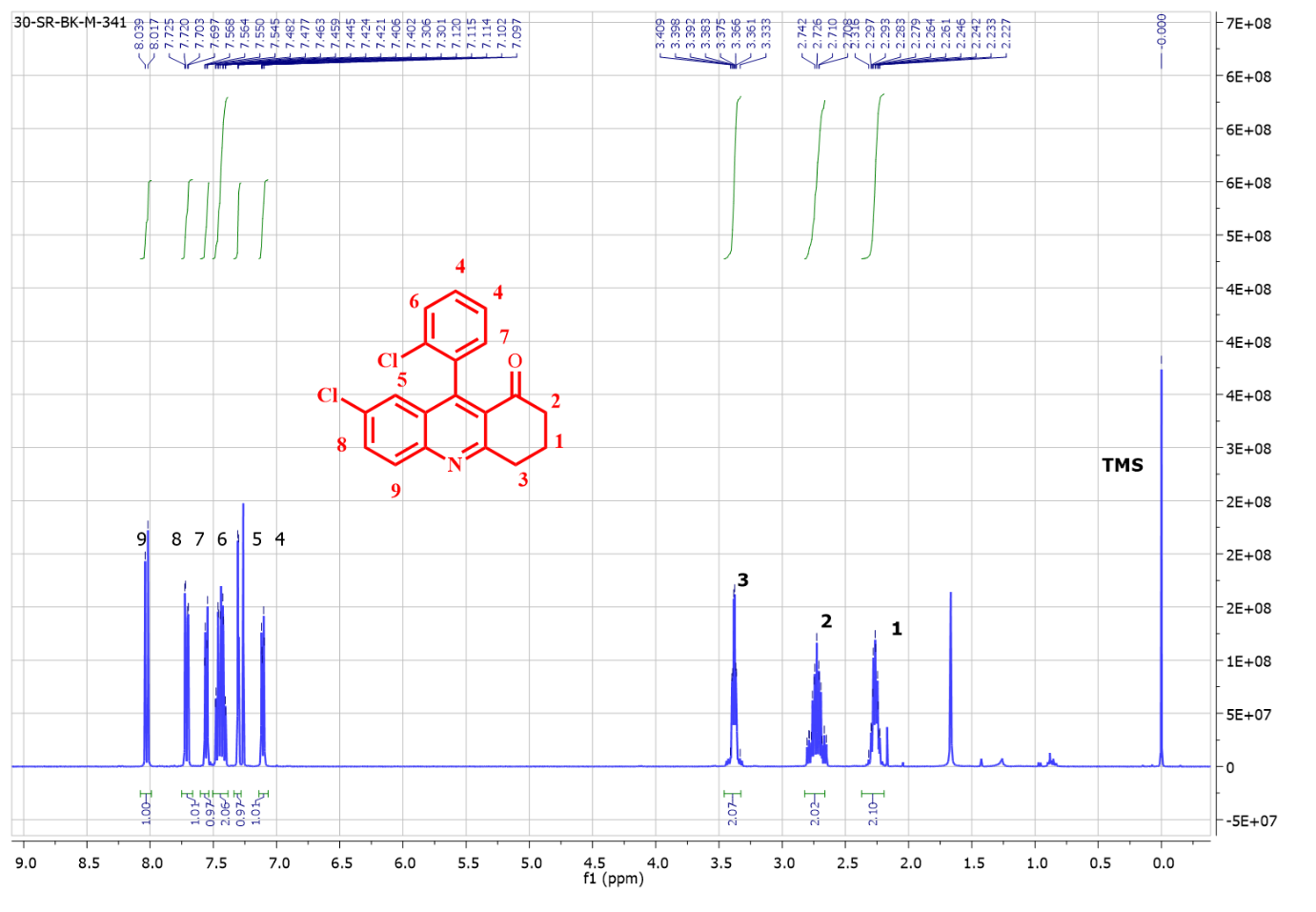


**Figure S12.** Mass spectrum 7-chloro-9-(2-chlorophenyl)-1,2,3,4-tetrahydroacridine &^1^HNMR of 7-chloro-9-(2-chlorophenyl)-3,4-dihydroacridin-1(2H)-one.


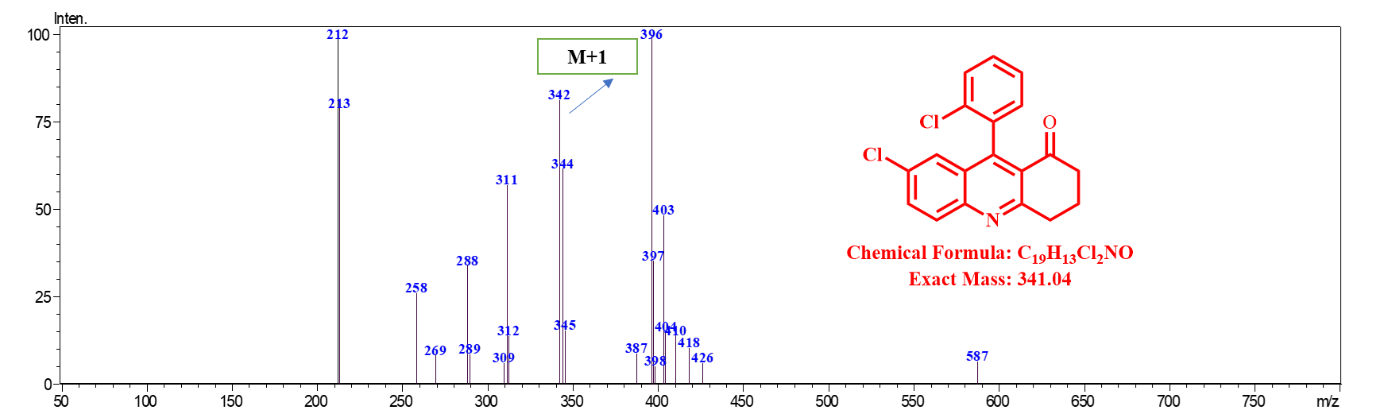

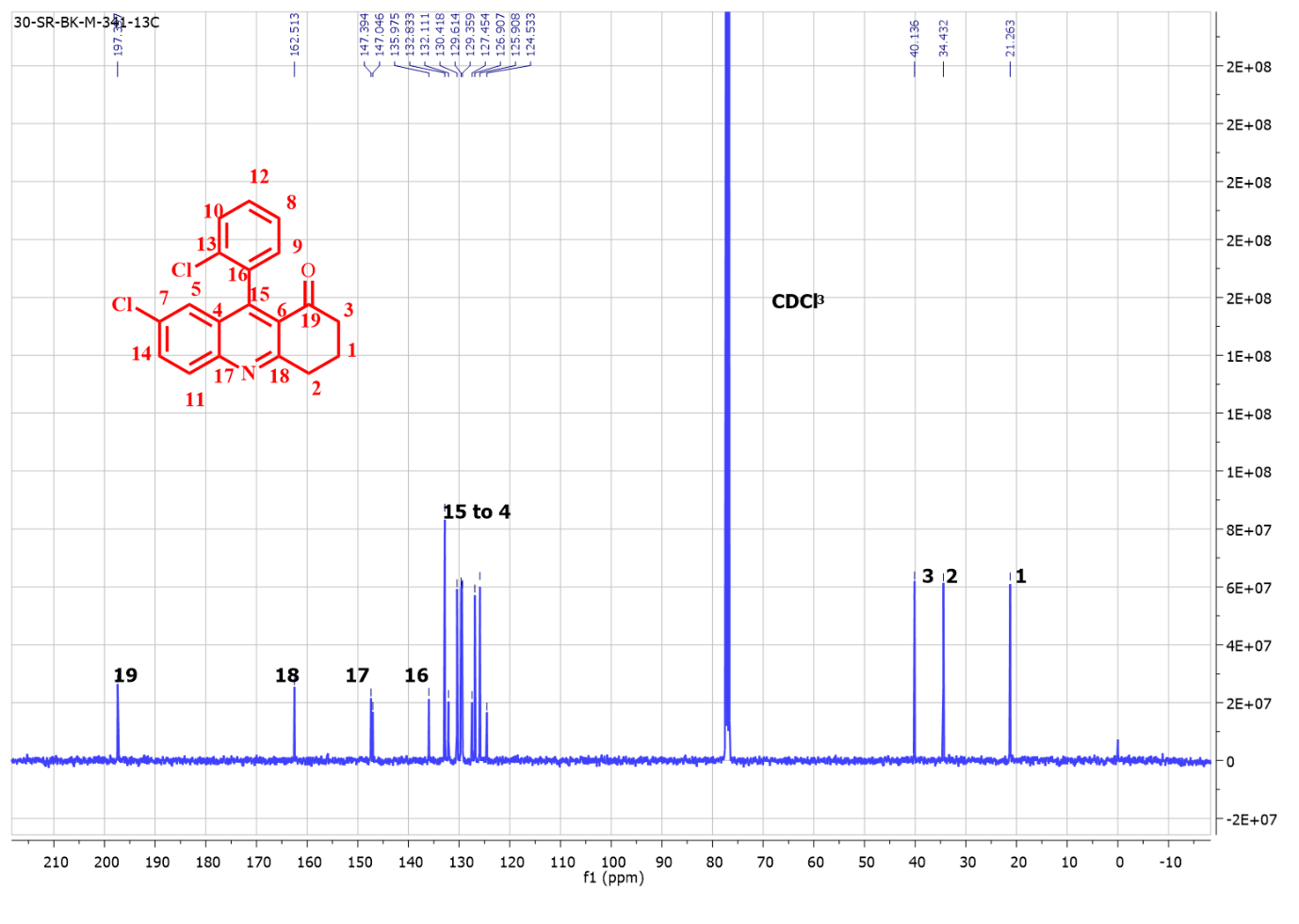


**Figure S13.**^13^CNMR and Mass spectrum of 7-chloro-9-(2-chlorophenyl)-3,4-dihydroacridin-1(2H)-one.

**
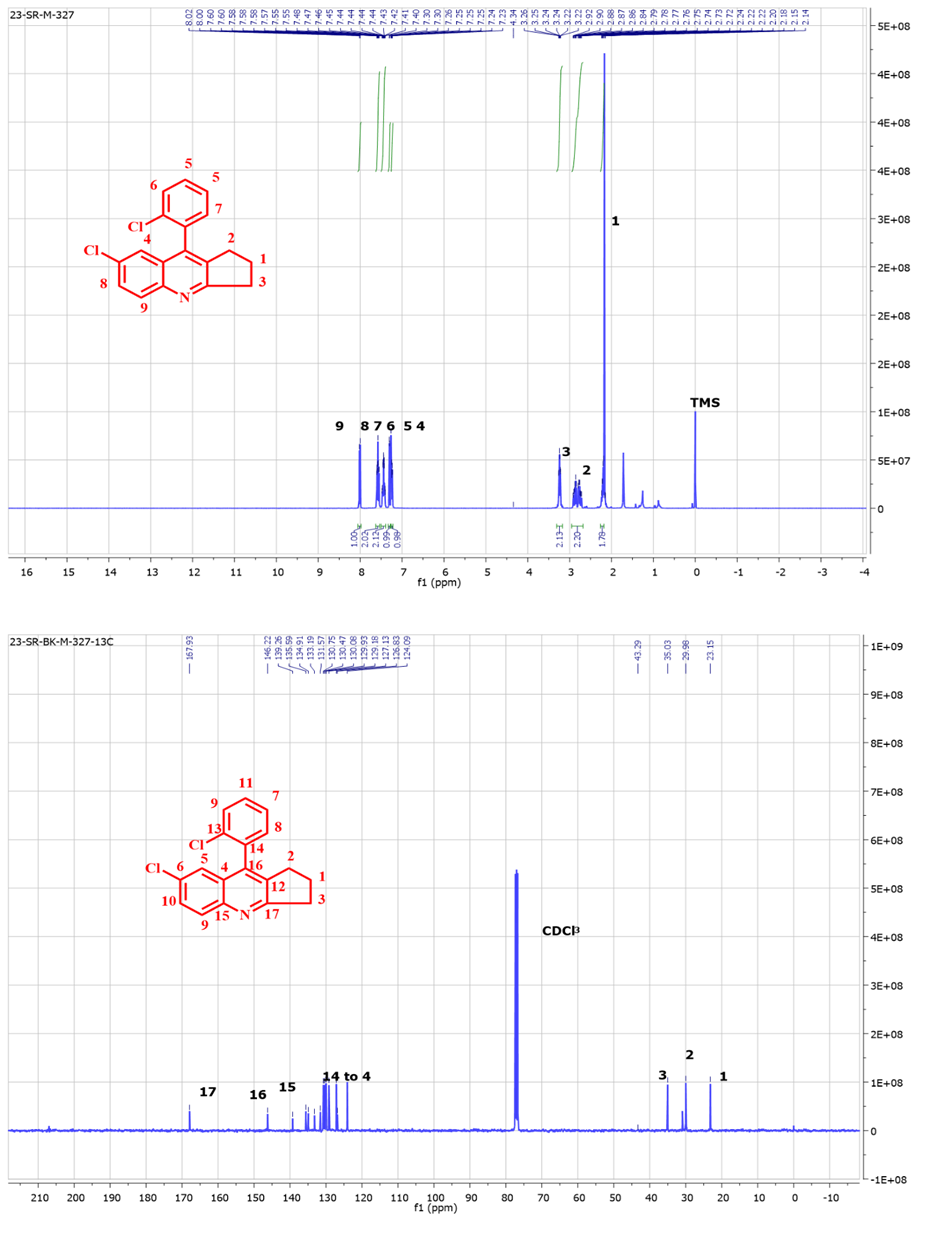
**

**Figure S14.** ^1^HNMR and ^13^CNMR 7-chloro-9-(2-chlorophenyl)-2,3-dihydro-1H-cyclopenta [b]quinoline.


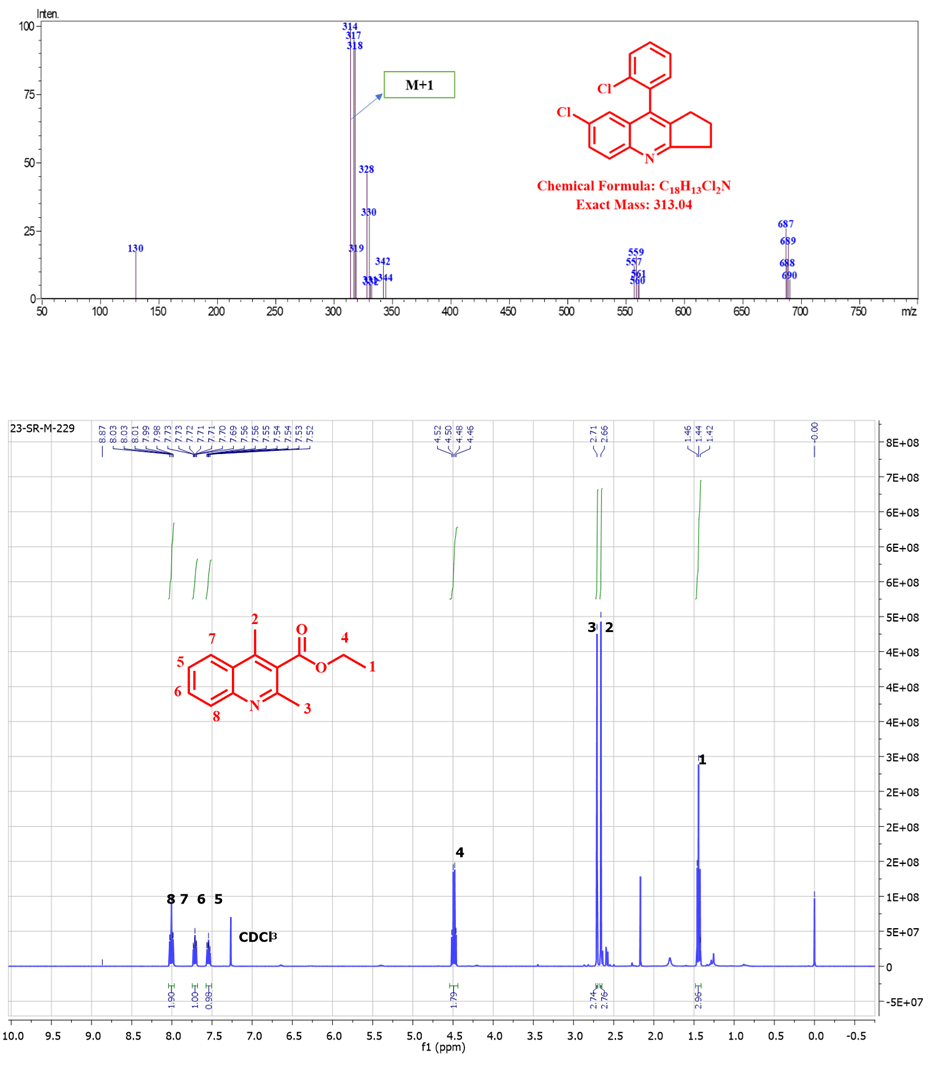


**Figure S15.** Massspectrum7-chloro-9-(2-chlorophenyl)-2,3-dihydro-1H-cyclopenta [b]quinoline and ^1^HNMR Ethyl 2,4-dimethylquinoline-3-carboxylate.


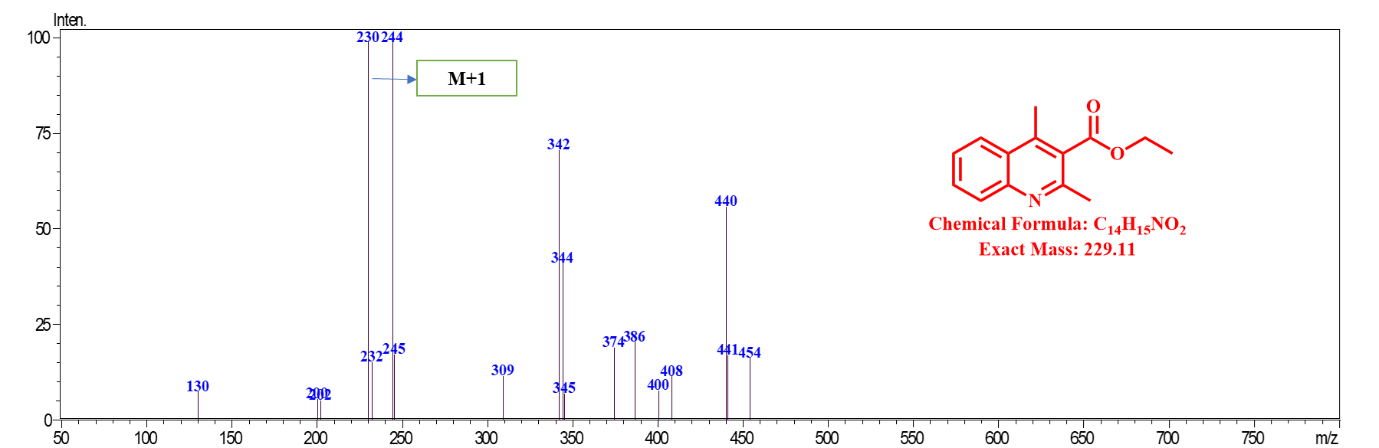

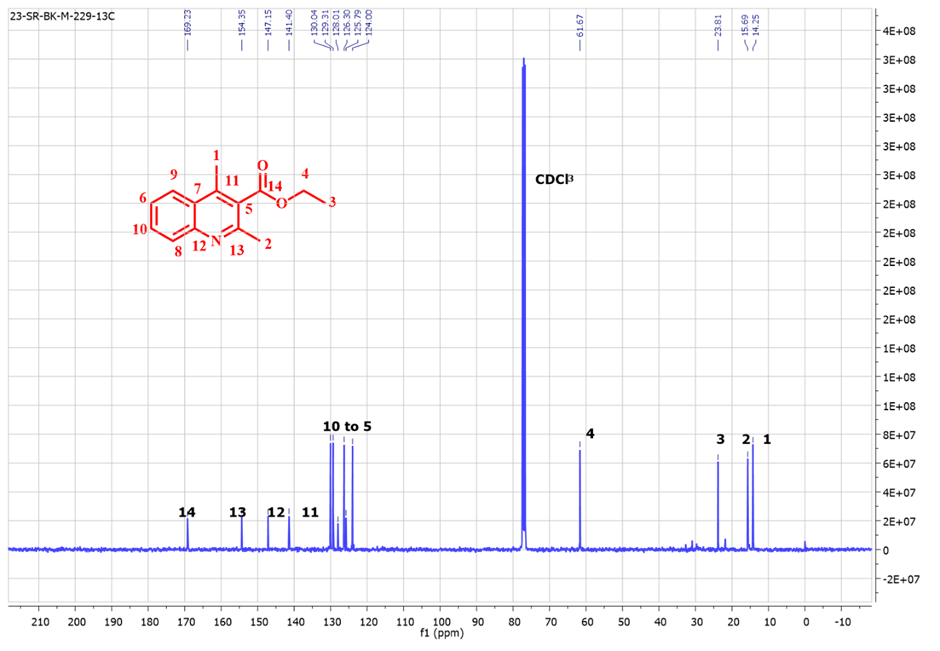


**Figure S16.** ^13^CNMR and mass spectrum Ethyl 2,4-dimethylquinoline-3-carboxylate.


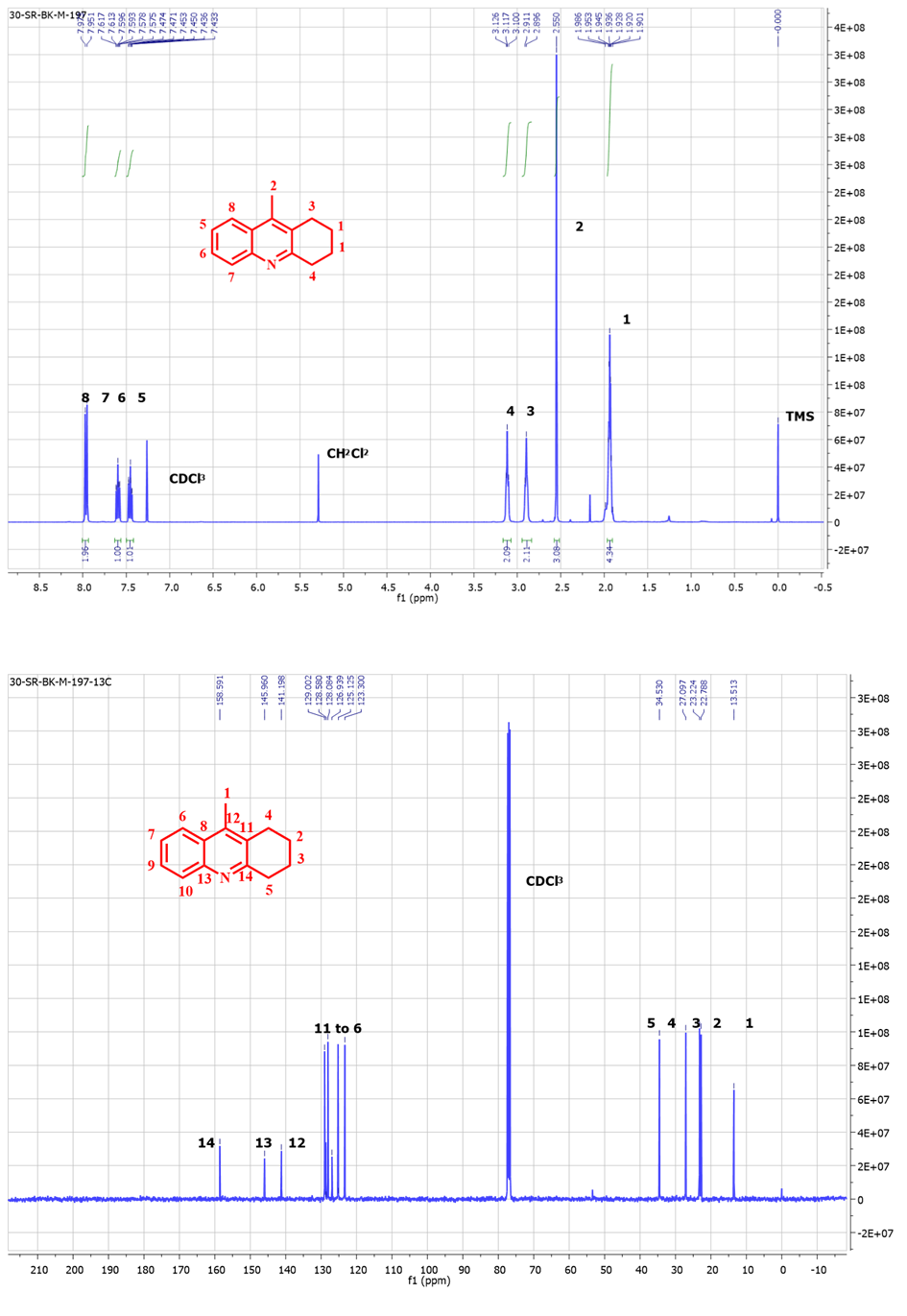


**Figure S17.** ^1^HNMR and ^13^ CNMR 9-methyl-1,2,3,4-tetrahydroacridine.


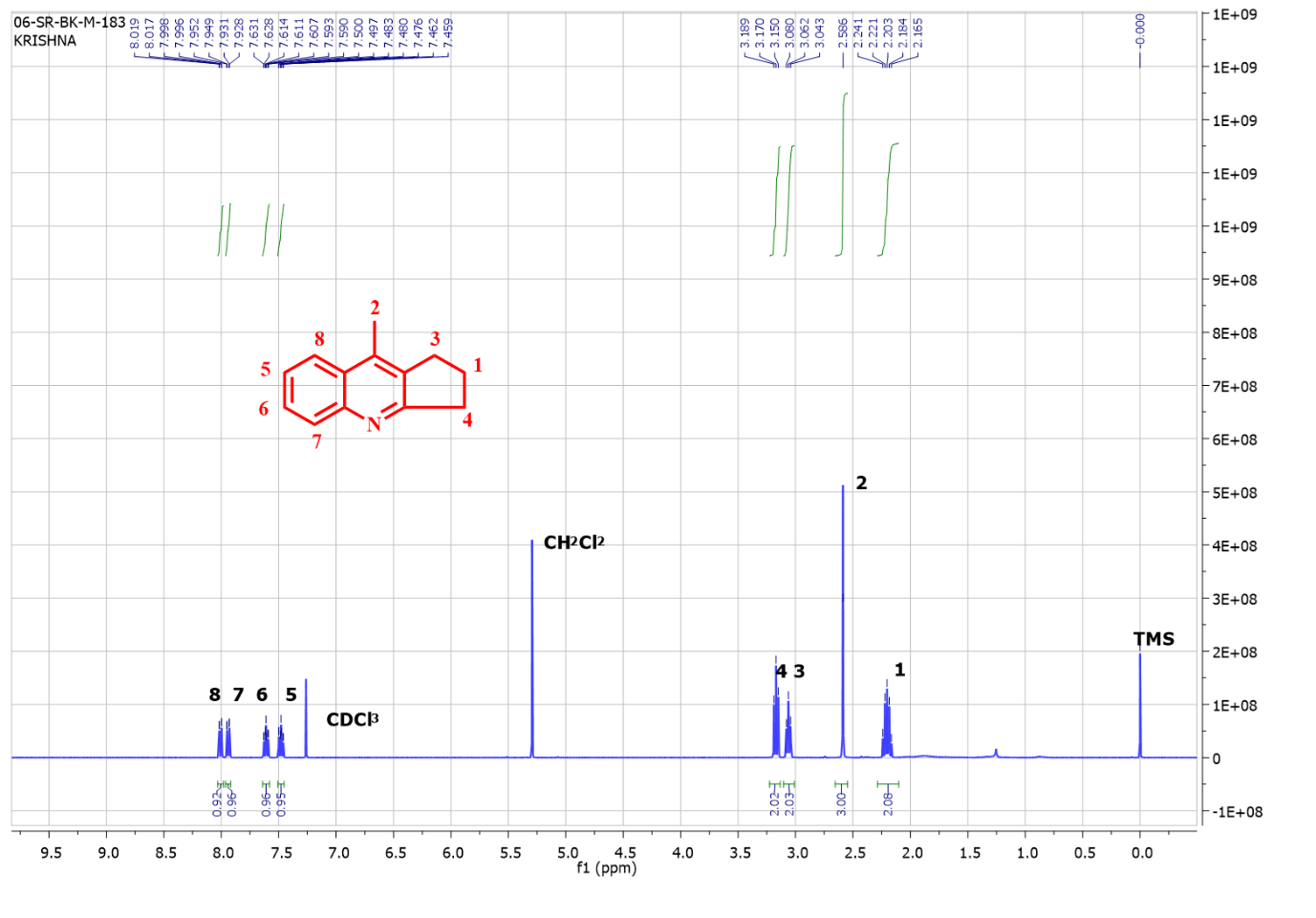

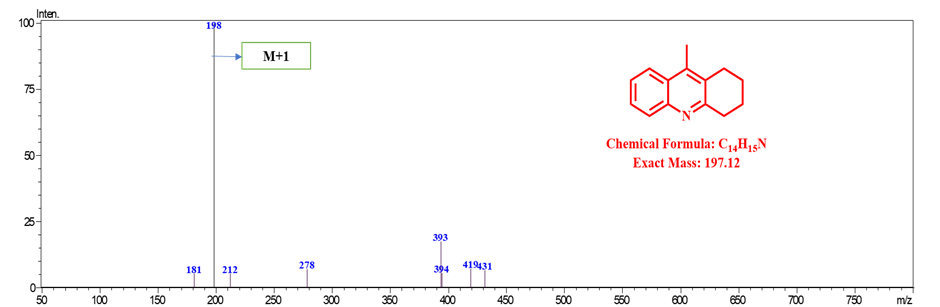


**Figure S18.** Mass spectrum 9-methyl-1,2,3,4-tetrahydroacridine and ^1^HNMR 9-methyl-2,3-dihydro-1H-cyclopenta[b]quinoline


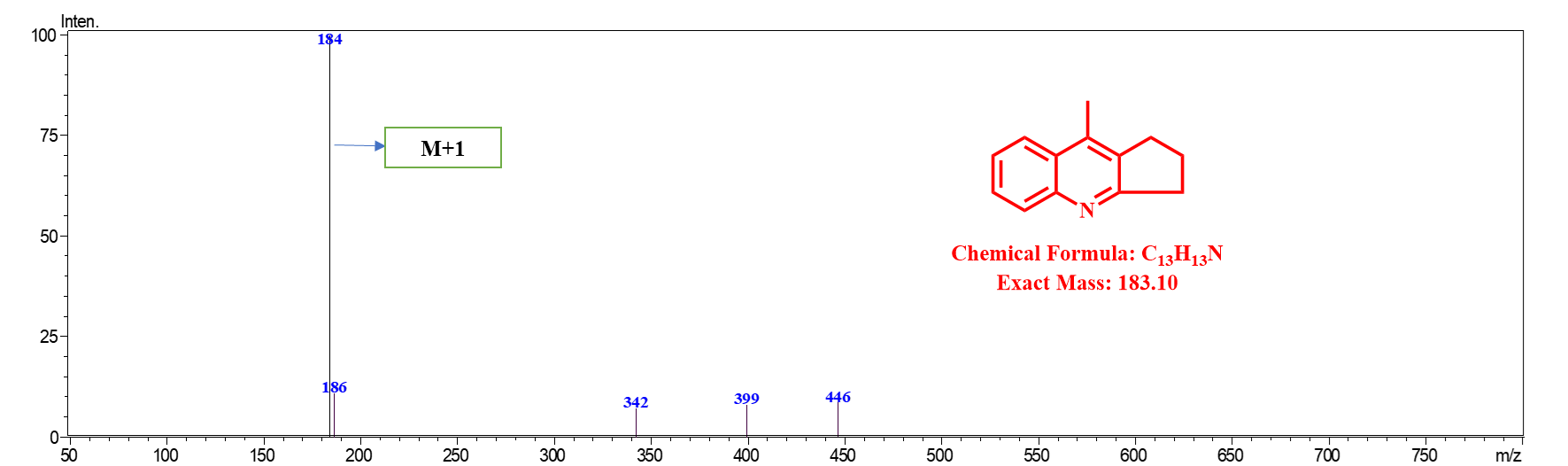

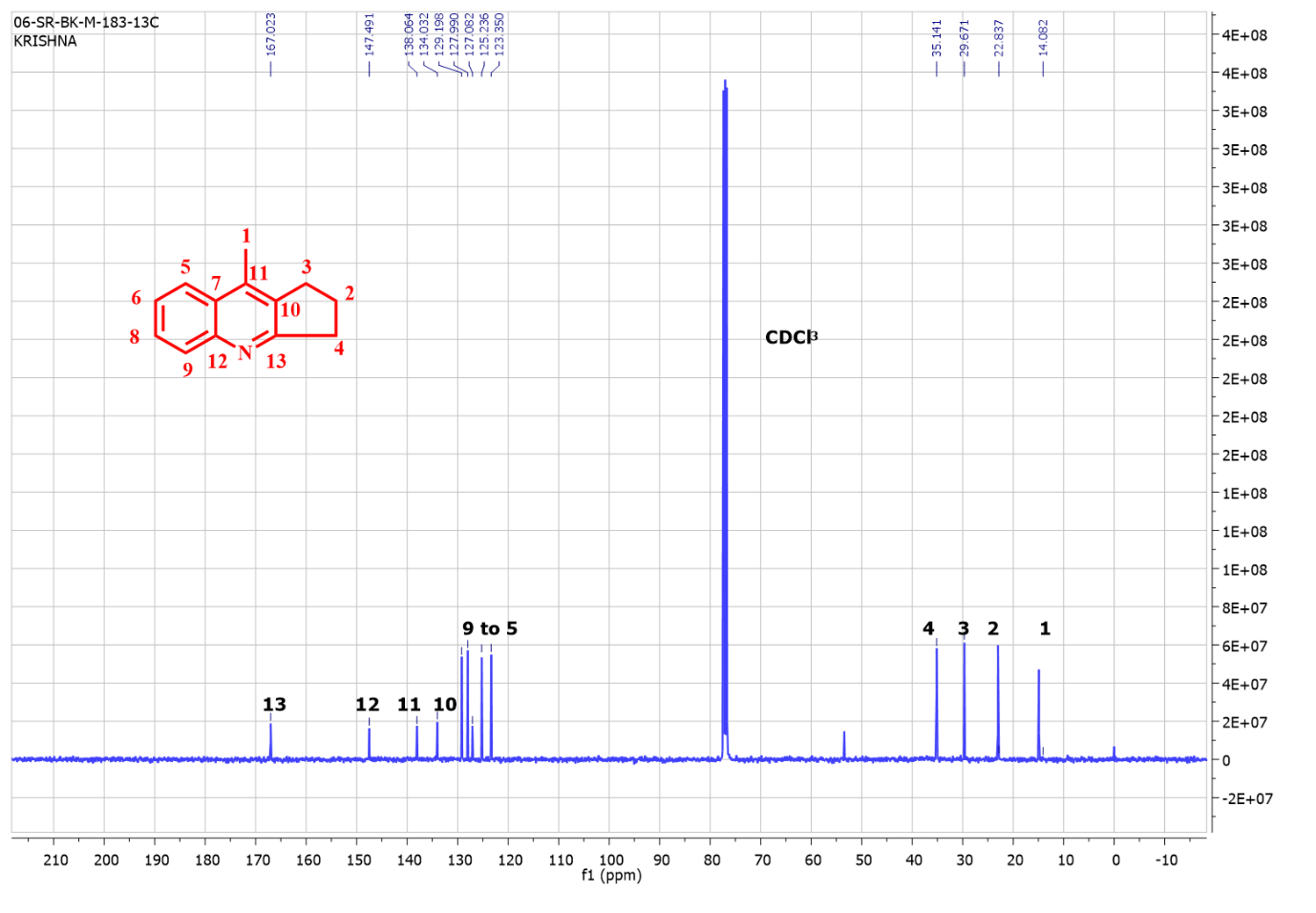


**Figure S19.** ^13^CNMR and mass spectrum of 9-methyl-2,3-dihydro-1H cyclopenta [b]quinoline.


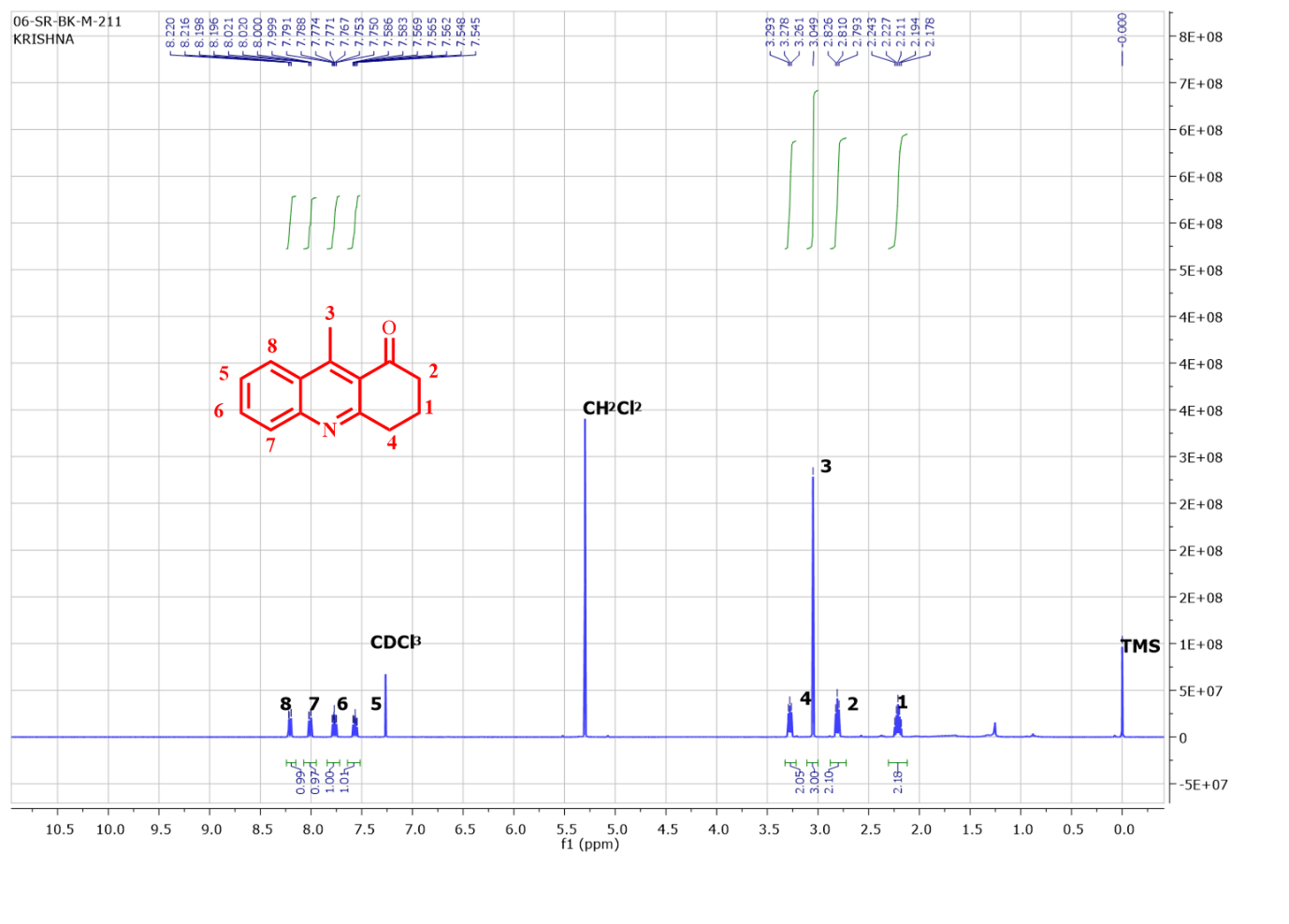

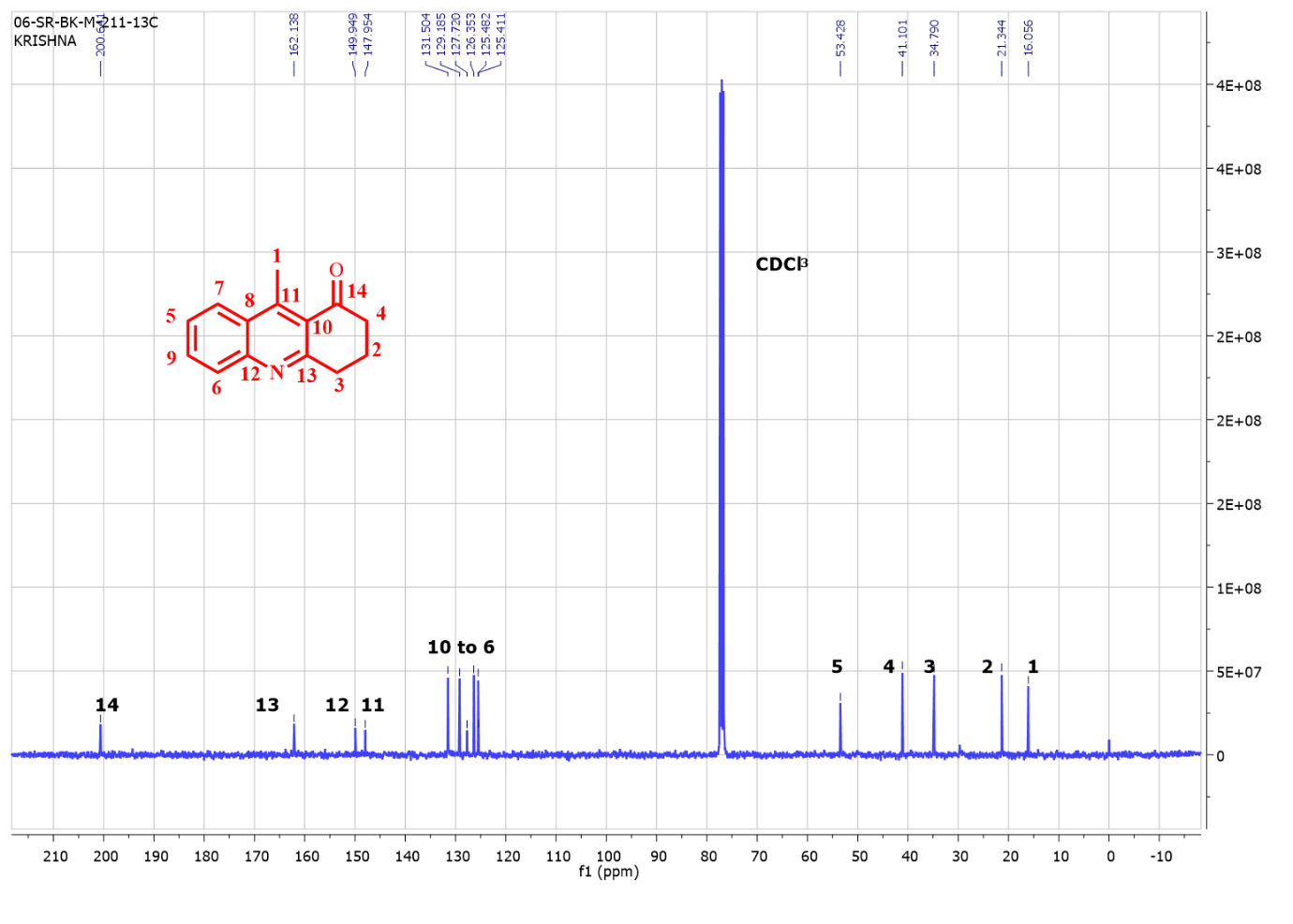


**Figure S20.** Mass spectrum 9-methyl-2,3-dihydro-1H-cyclopenta[b]quinoline & ^1^HNMR 9-methyl-3,4-dihydroacridin-1(2H)-one.


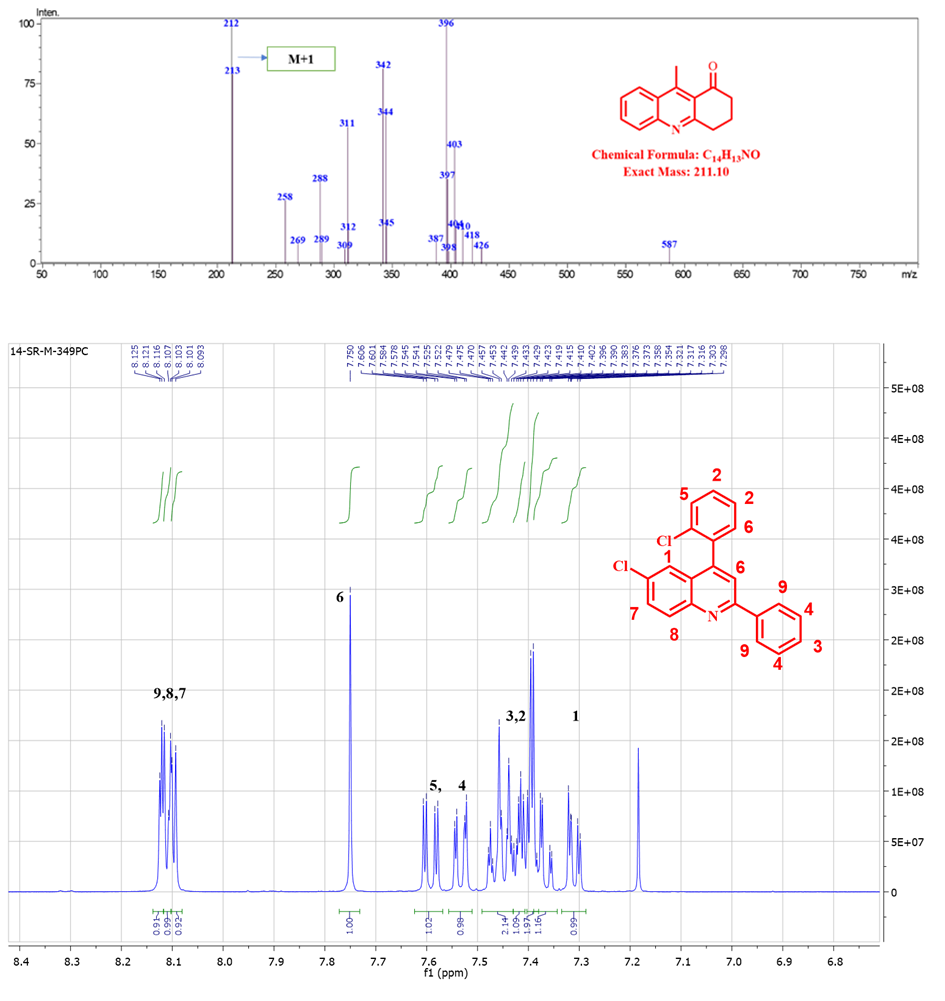


**Figure S21.** Mass spectrum 9-methyl-3,4-dihydroacridin-1(2H)-one &^1^HNMR of 6-chloro-4-(2-chlorophenyl)-2-phenylquinoline.


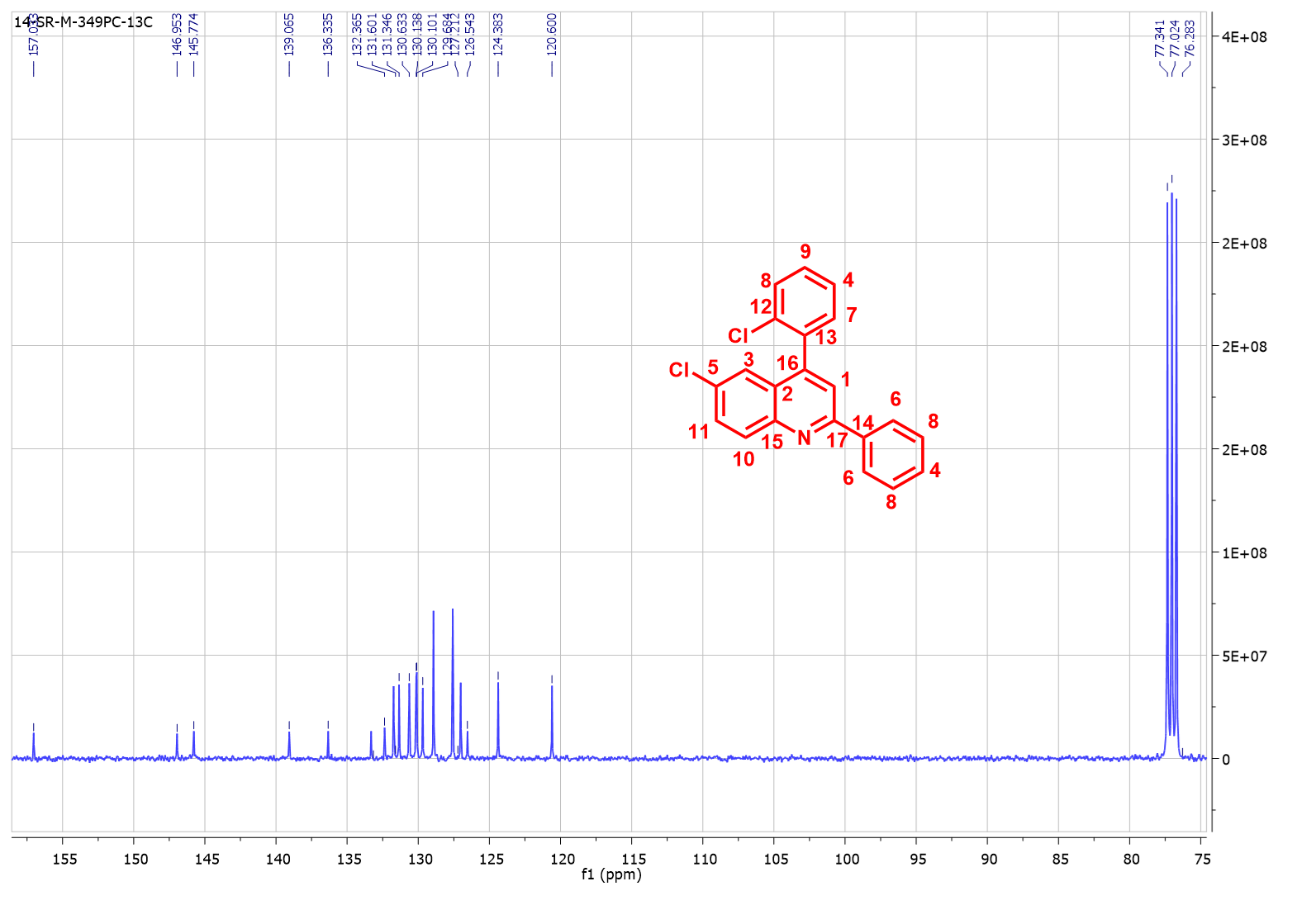

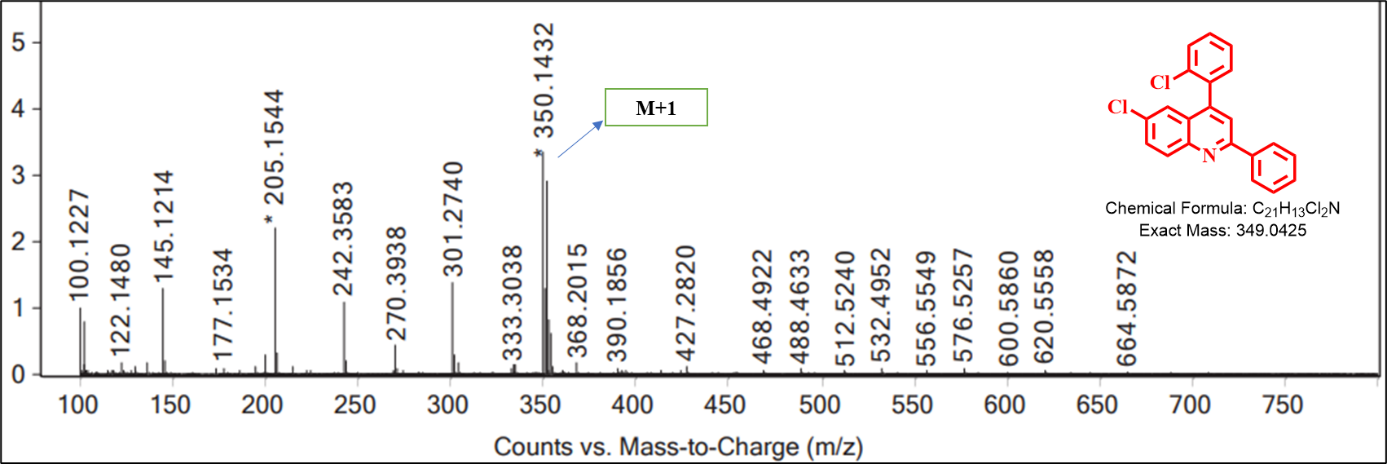


**Figure S22.** ^13^CNMR & Mass spectrum of 6-chloro-4-(2-chlorophenyl)-2-phenylquinoline.


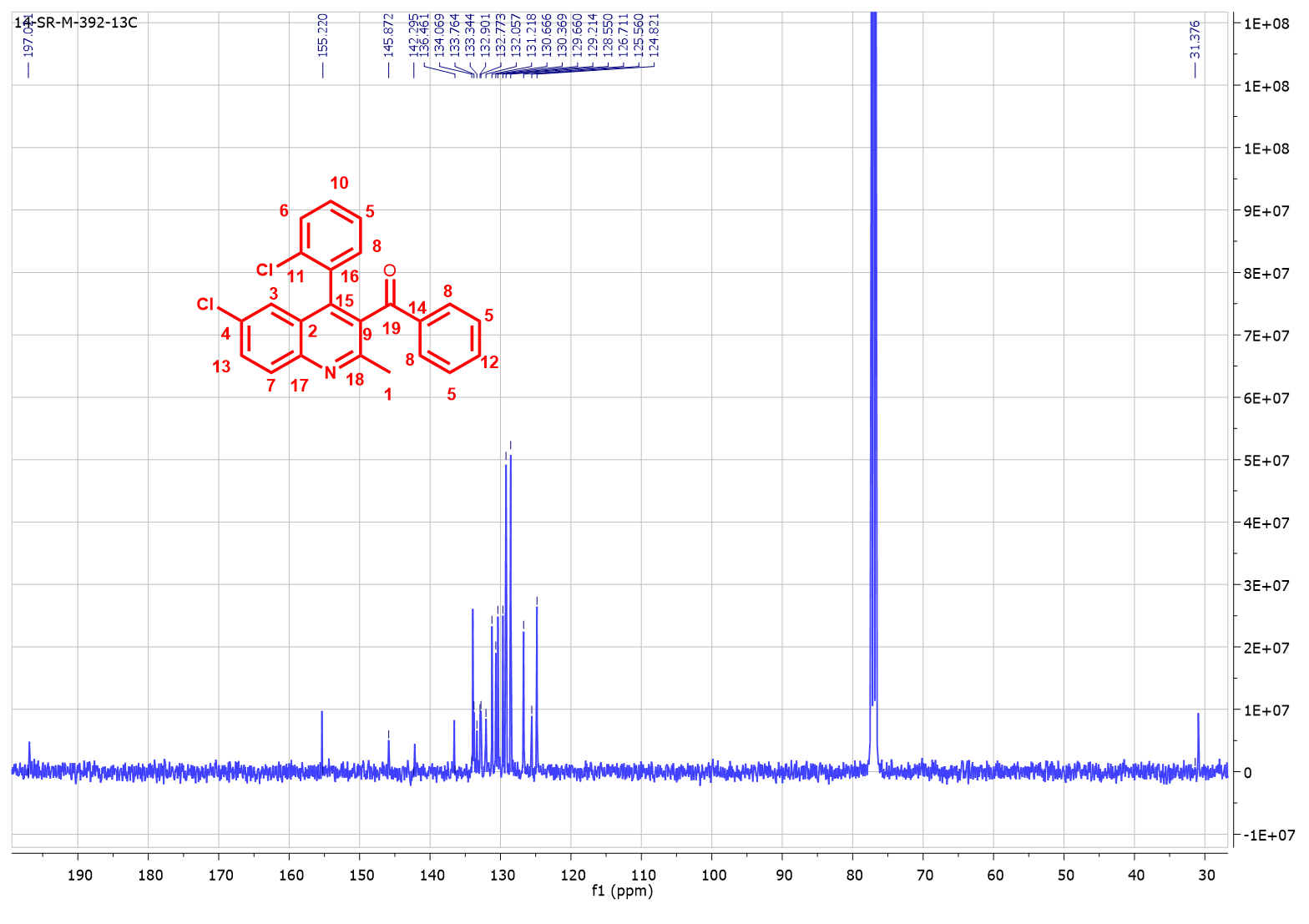

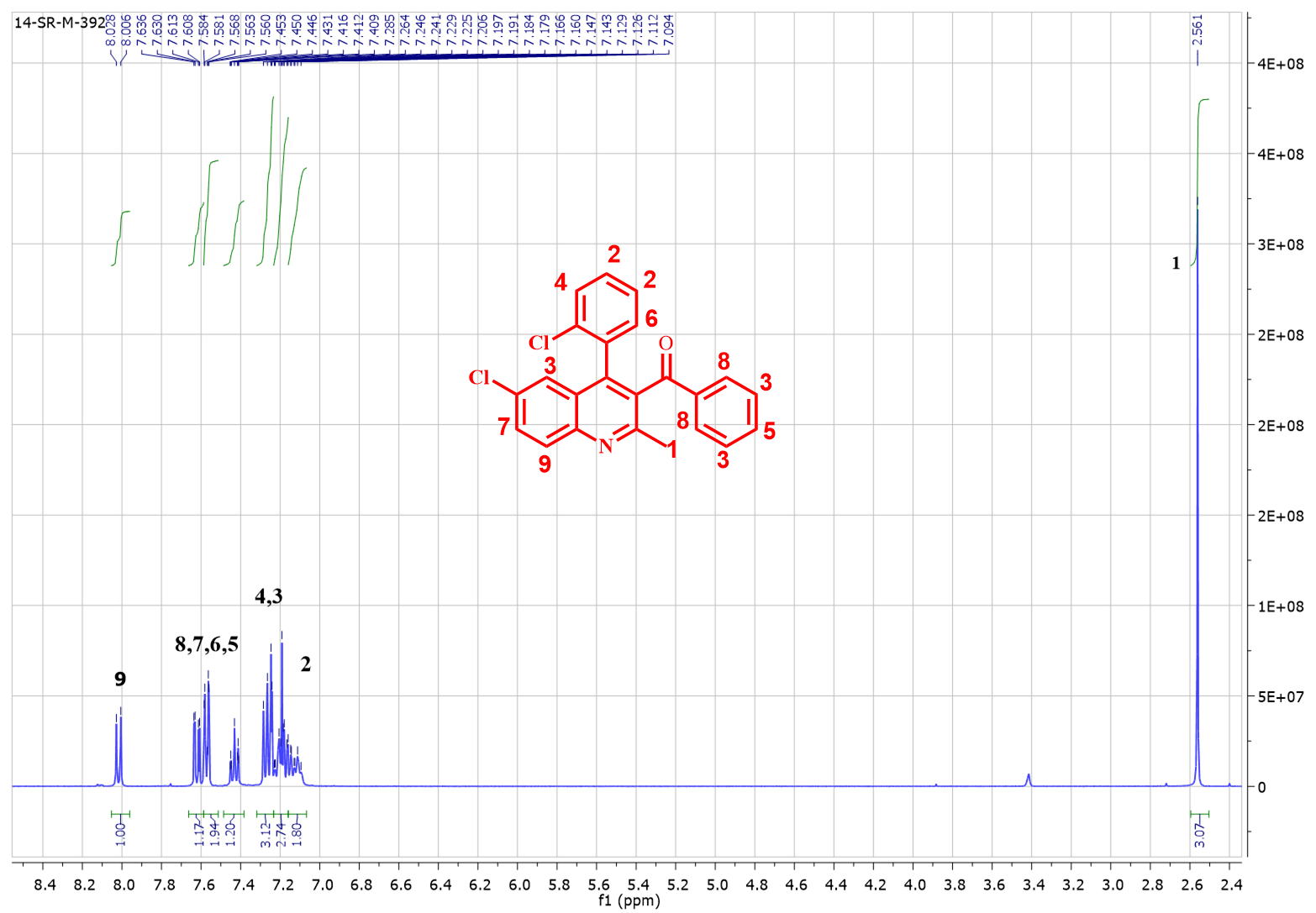


**Figure S23.** ^1^HNMR and ^13^ CNMR of 1-(6-chloro-4-(2-chlorophenyl)-2-phenylquinolin-3-yl)ethan-1-one.


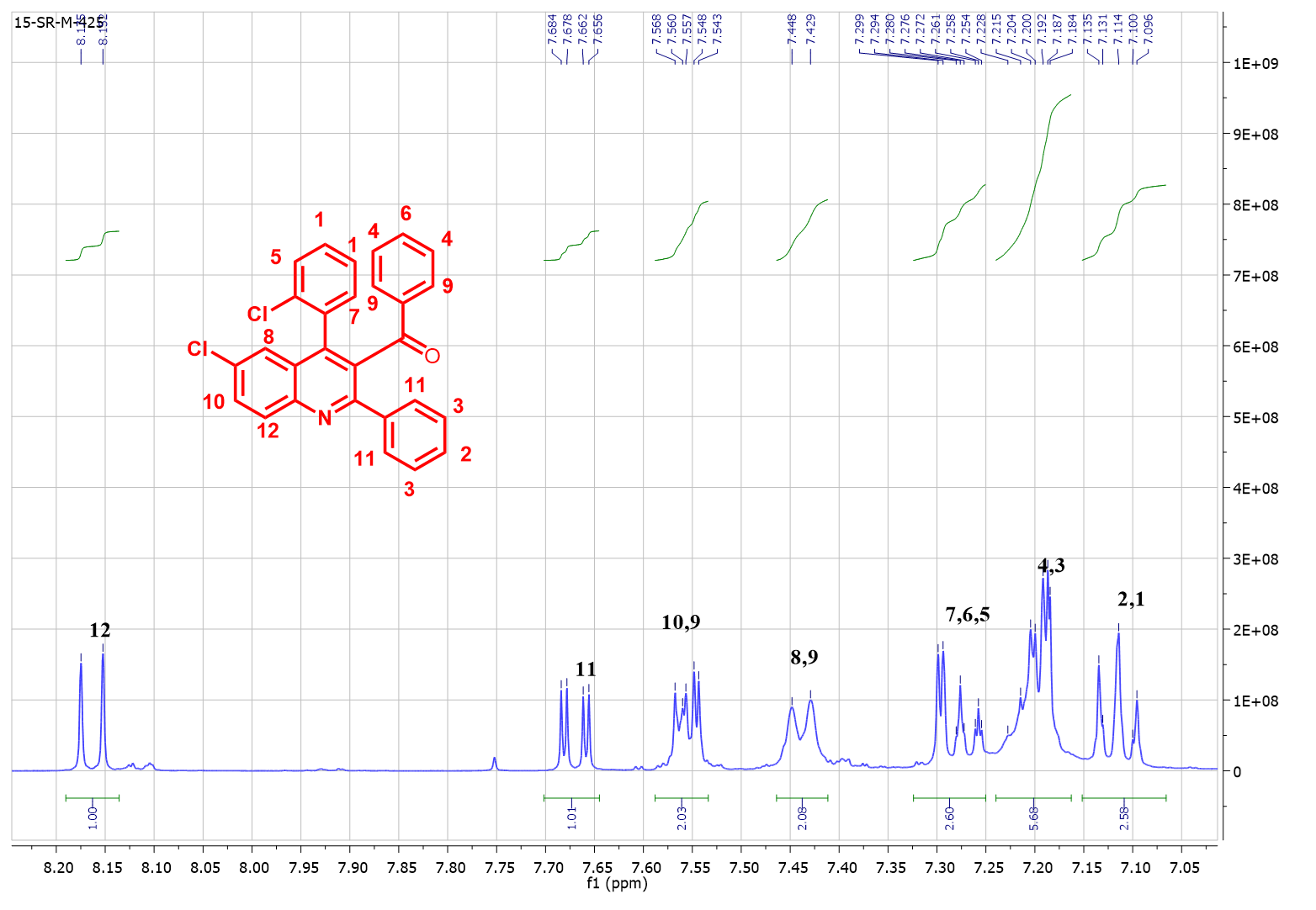

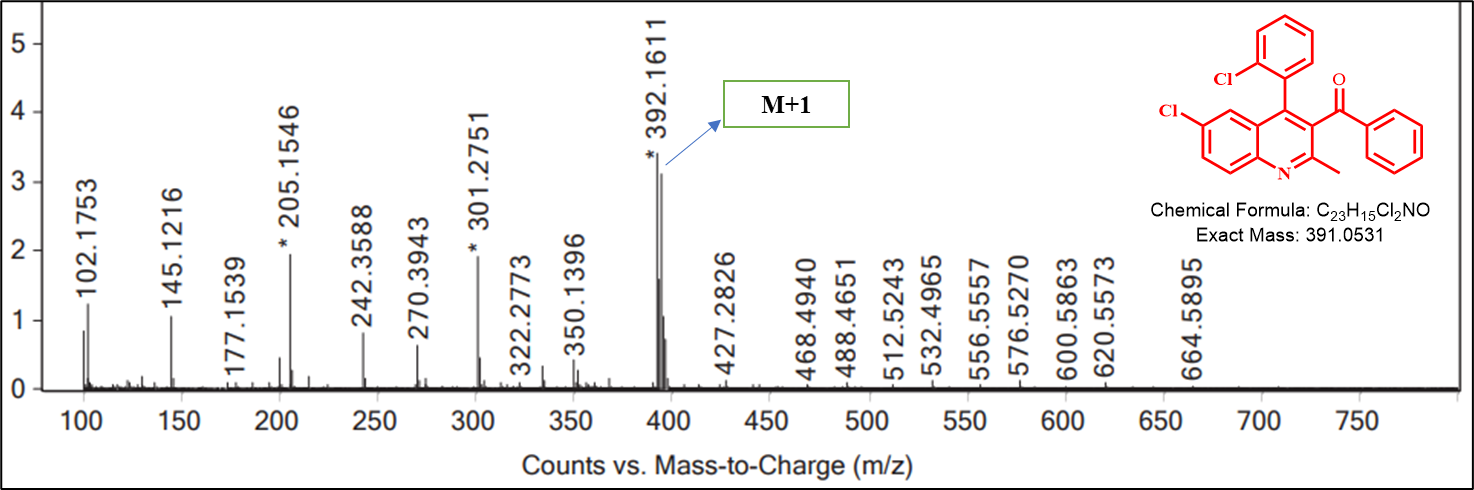


**Figure S24.** Mass spectrum of 1-(6-chloro-4-(2-chlorophenyl)-2-phenylquinolin-3-yl)ethan-1-one &^1^HNMR of (6-chloro-4-(2-chlorophenyl)-2-phenylquinolin-3-yl)(phenyl)methanone.


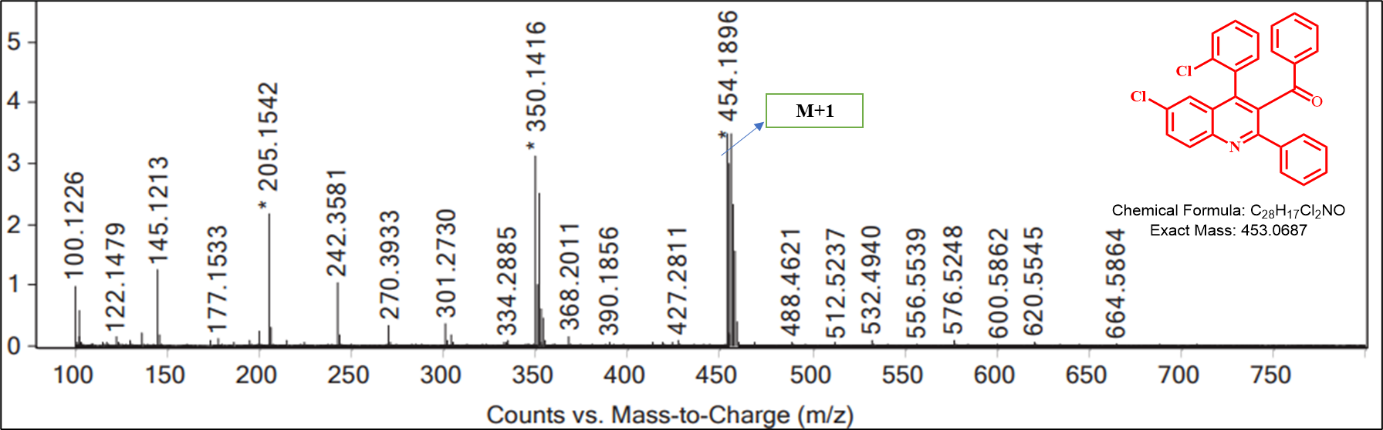

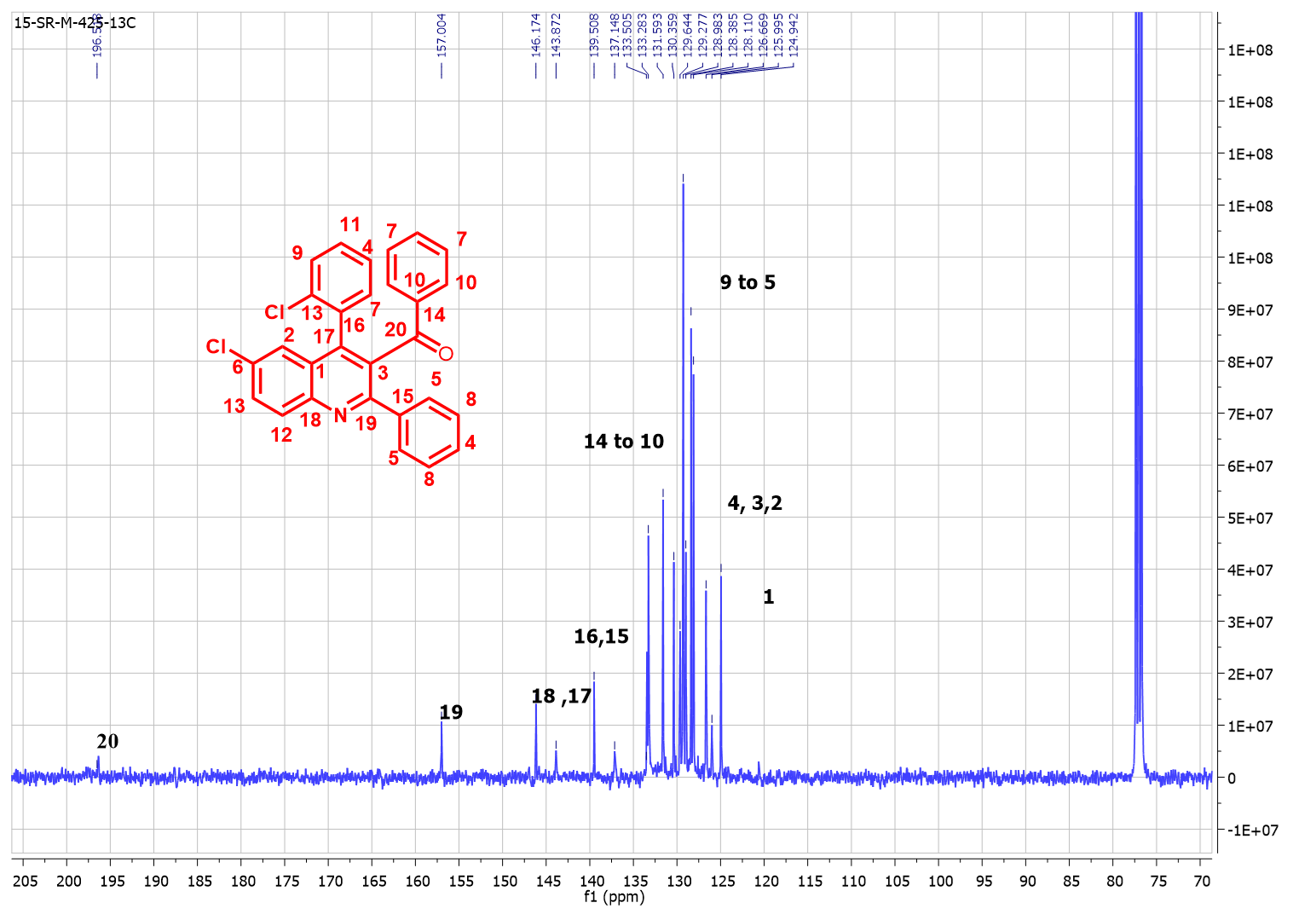


**Figure S25.**^13^CNMR and mass spectrum of (6-chloro-4-(2-chlorophenyl)-2-phenylquinolin-3-yl)(phenyl)methanone.


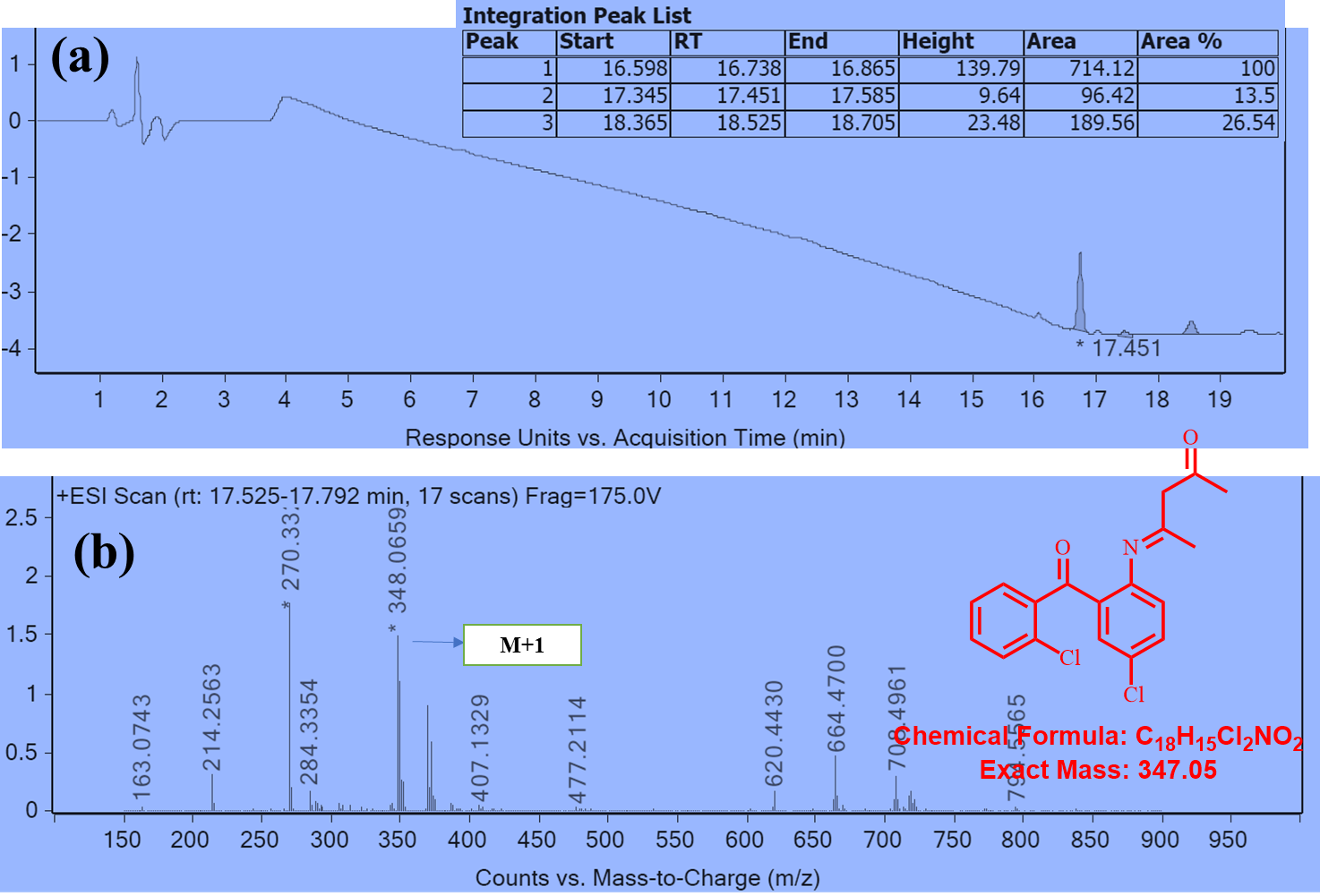


**Figure S26.** (a) HPLC chromatogram, (b) LCMS of 4-((4-chloro-2-(2-chlorobenzoyl) phenyl) imino) pentan-2-one.

**^1^H NMR,^13^C NMR and Mass** **spectrum results of products.**

**1-(2-methylquinolin-3-yl)ethan-1-one**: Pale yellow solid, yield: 77 %, 143 mg, ^1^H NMR (400 MHz, CDCl_3_) δ 8.40 (s, 1H), 7.96 (dd, *J* = 8.5, 0.7 Hz, 1H), 7.85 – 7.75 (m, 1H), 7.75 – 7.60 (m, 1H), 7.48 (tt, *J* = 9.5, 1.8 Hz, 1H), 7.19 (s, 1H), 2.84 (s, 3H), 2.64 (s, 3H); ^13^C NMR (101 MHz, CDCl_3_) δ 199.95, 157.50, 148.27, 138.21, 131.64, 130.98, 128.57, 126.37, 125.63, 29.42, 25.77. LCMS (ESI/TOF-Q): m/z = (M + H) 185.1, Calculated value C_12_H_11_NO 186.16.

**1-(2,4-dimethylquinolin-3-yl)ethan-1-one**: Brown solid, yield: 86 %, 171 mg, ^1^H NMR (400 MHz, CDCl_3_) δ 8.02 (dd, *J* = 8.4, 0.6 Hz, 1H), 7.98 (dd, *J* = 8.4, 0.8 Hz, 1H), 7.74 – 7.71 (m, 1H), 7.69 (d, *J* = 1.3 Hz, 1H), 7.57 (d, *J* = 1.1 Hz, 1H), 7.56 – 7.52 (m, 1H), 2.64 (s, 3H), 2.60 (s, 3H), 2.59 (s, 3H);  ^13^C NMR (101 MHz, CDCl_3_) δ 206.68, 152.63, 146.99, 135.74, 129.83, 129.31, 126.40, 125.99, 123.67, 32.67, 23.58, 15.25. MS (ESI): m/z = (M + H) 200, Calculated value C_13_H_13_NO 199.10.

**1-(2-methyl-4-phenylquinolin-3-yl)ethan-1-one**: Pale yellow solid, yield: 93 %, 243 mg,  ^1^H NMR (400 MHz, CDCl_3_) δ 8.08 (d, *J* = 8.4 Hz, 1H), 7.76 – 7.68 (m, 1H), 7.62 (dd, *J* = 8.4, 1.0 Hz, 1H), 7.54 – 7.48 (m, 1H), 7.44 (t, *J* = 7.6 Hz, 1H), 7.39 – 7.33 (m, 1H), 2.70 (s, 1H), 2.00 (s, 1H); ^13^C NMR (101 MHz, CDCl_3_) δ 204.46, 154.08, 145.66, 140.49, 135.69, 133.40, 132.80, 132.04, 131.19, 130.87, 130.64, 130.06, 127.42, 125.43, 124.59, 31.29, 24.04, LCMS (ESI/TOF-Q): m/z = (M + H) 262.09, Calculated value C_18_H_15_NO 261.1105.

**1-(2-methyl-6-nitro-4-phenylquinolin-3-yl)ethan-1-one:** Yellow solid, yield: 89 %, 273 mg, ^1^H NMR (400 MHz, CDCl_3_) δ 8.58 (d, *J* = 2.3 Hz, 1H), 8.51 – 8.46 (m, 1H), 8.20 (d, *J* = 9.2 Hz, 1H), 7.61 – 7.56 (m, 3H), 7.39 – 7.34 (m, 2H), 2.74 (s, 3H), 2.02 (s, 3H);  ^13^C NMR (101 MHz, CDCl_3_) δ 204.34, 157.97, 149.58, 145.68, 136.28, 130.79, 129.92, 129.86, 129.27, 124.41, 123.61, 123.19, 31.71, 24.05. MS (ESI): m/z = (M + H) 307.05, Calculated value C_13_H_14_N_2_O_3_ 306.10.

**1-(6-chloro-4-(2-chlorophenyl)-2-methylquinolin-3-yl)ethan-1-one**: Pale yellow solid, yield: 97 %, 320 mg, ^1^H NMR (400 MHz, CDCl_3_) δ 8.02 (d, *J* = 9.0 Hz, 1H), 7.66 (dd, *J* = 9.0, 2.3 Hz, 1H), 7.59 (dd, *J* = 8.0, 1.2 Hz, 1H), 7.50 (ddd, *J* = 9.6, 6.9, 2.9 Hz, 1H), 7.46 – 7.39 (m, 1H), 7.27 (d, *J* = 1.9 Hz, 1H), 7.24 (dd, *J* = 7.5, 1.7 Hz, 1H), 2.70 (s, 3H), 2.21 – 2.12 (m, 3H); ^13^C NMR (101 MHz, CDCl_3_) δ 204.46, 154.08, 145.66, 140.49, 135.69, 133.40, 132.80, 132.04, 131.19, 130.87, 130.64, 130.06, 127.42, 125.43, 124.59, 31.29, 24.04, LCMS (ESI/TOF-Q): m/z = (M + H) 330.05, Calculated value C_18_H_13_Cl_2_NO 329.09.

**Ethyl 6-chloro-4-(2-chlorophenyl)-2-methylquinoline-3-carboxylate:** Pale yellow solid, yield: 90 %, 324 mg,  ^1^H NMR (400 MHz, CDCl_3_) δ 8.03 (d, *J* = 9.0 Hz, 1H), 7.66 (dd, *J* = 9.0, 2.3 Hz, 1H), 7.56 (dd, *J* = 8.0, 0.9 Hz, 1H), 7.46 (td, *J* = 7.8, 1.6 Hz, 1H), 7.39 (td, *J* = 7.5, 1.1 Hz, 1H), 7.28 (d, *J* = 2.2 Hz, 1H), 7.24 (d, *J* = 1.6 Hz, 1H), 4.14 – 4.02 (m, 2H), 2.81 (s, 3H), 0.97 (t, *J* = 7.1 Hz, 3H). ^13^C NMR (101 MHz, CDCl_3_) δ 167.44, 155.56, 145.91, 143.22, 134.19, 133.71, 132.68, 131.43, 130.95, 130.65, 130.33, 129.69, 128.01, 126.79, 125.53, 124.86, 61.49, 24.01, 13.58. MS (ESI): m/z = (M + H) 360.10, Calculated value C_19_H_15_Cl_2_NO_2_ 359.06.

**7-chloro-9-(2-chlorophenyl)-1,2,3,4-tetrahydroacridine:** Pale yellow solid, yield: 95 %, 310 mg, ^1^H NMR (400 MHz, CDCl_3_) δ 7.97 (d, *J* = 9.0 Hz, 1H), 7.61 – 7.51 (m, 2H), 7.44 (pd, *J* = 7.5, 2.0 Hz, 2H), 7.17 (dt, *J* = 5.8, 3.2 Hz, 1H), 7.13 (d, *J* = 2.3 Hz, 1H), 3.24 – 3.14 (m, 2H), 2.60 – 2.46 (m, 2H), 2.03 – 1.93 (m, 2H), 1.85 – 1.76 (m, 2H); ^13^C NMR (101 MHz, CDCl_3_) δ 159.60, 144.71, 142.91, 135.25, 133.26, 131.50, 130.66, 130.24, 130.06, 129.83, 129.43, 127.33, 126.80, 123.84, 34.16, 30.93, 27.46, 22.69.MS (ESI): m/z = (M + H) 328, Calculated value C_19_H_15_Cl_2_N 327.06.

**7-chloro-9-(2-chlorophenyl)-3,4-dihydroacridin-1(2H)-one:** Light brown solid, yield: 96 %, 328 mg, ^1^H NMR (400 MHz, CDCl_3_) δ 8.03 (d, *J* = 9.0 Hz, 1H), 7.71 (dd, *J* = 9.0, 2.3 Hz, 1H), 7.56 (dd, *J* = 7.5, 1.8 Hz, 1H), 7.49 – 7.39 (m, 2H), 7.30 (d, *J* = 2.3 Hz, 1H), 7.15 – 7.07 (m, 1H), 3.45 – 3.31 (m, 2H), 2.82 – 2.64 (m, 2H), 2.33 – 2.20 (m, 2H);  ^13^C NMR (101 MHz, CDCl_3_) δ 197.40, 162.51, 147.39, 147.05, 135.97, 132.83, 132.11, 130.42, 129.61, 129.36, 127.45, 126.91, 125.91, 124.53, 40.14, 34.43, 21.26., MS (ESI): m/z = (M + H) 342, Calculated value C_19_H_13_Cl_2_NO_2_ 341.04.

**7-chloro-9-(2-chlorophenyl)-2,3-dihydro-1H-cyclopenta [b]quinoline:** Pale yellow solid, yield: 89 %, 279 mg, ^1^H NMR (400 MHz, CDCl_3_) δ 8.01 (d, *J* = 8.9 Hz, 1H), 7.58 (ddd, *J* = 11.6, 8.0, 2.1 Hz, 2H), 7.49 – 7.39 (m, 2H), 7.30 (d, *J* = 2.3 Hz, 1H), 7.26 – 7.22 (m, 1H), 3.24 (td, *J* = 8.0, 2.7 Hz, 2H), 2.94 – 2.71 (m, 2H), 2.31 – 2.16 (m, 5H); ^13^C NMR (101 MHz, CDCl_3_) δ 167.93, 146.22, 139.26, 135.59, 134.91, 133.19, 131.57, 130.75, 130.47, 130.08, 129.93, 129.18, 127.13, 126.83, 124.09, 43.29, 35.03, 29.98, 23.15, MS (ESI): m/z = (M + H) 314, Calculated value C_18_H_13_Cl_2_N 313.04.

**Ethyl 2,4-dimethylquinoline-3-carboxylate**: Light brown oil, yield: 80 %, 184 mg, ^1^H NMR (400 MHz, CDCl_3_) δ 8.04 – 7.97 (m, 1H), 7.71 (ddd, *J* = 8.4, 6.9, 1.4 Hz, 1H), 7.54 (ddd, *J* = 8.2, 6.9, 1.3 Hz, 1H), 4.49 (q, *J* = 7.1 Hz, 1H), 2.71 (s, 1H), 2.66 (s, 1H), 1.44 (t, *J* = 8.0 Hz, 2H); ^13^C NMR (101 MHz, CDCl_3_) δ 169.23, 154.35, 147.15, 141.40, 130.04, 129.31, 128.01, 126.30, 125.79, 124.00, 61.67, 23.81, 15.69, 14.25. MS (ESI): m/z = (M + H) 230.02, Calculated value C_14_H_15_NO_2_ 229.11.

**9-methyl-1,2,3,4-tetrahydroacridine:** Light brown solid, yield: 94 %, 185 mg, ^1^H NMR (400 MHz, CDCl_3_) δ 7.96 (d, *J* = 8.8 Hz, 1H), 7.63 – 7.56 (m, 1H), 7.49 – 7.42 (m, 1H), 3.11 (t, *J* = 5.2 Hz, 1H), 2.90 (d, *J* = 5.9 Hz, 1H), 2.55 (s, 2H), 2.02 – 1.88 (m, 3H); ^13^C NMR (101 MHz, CDCl_3_) δ 158.59, 145.96, 141.20, 129.00, 128.58, 128.08, 126.94, 125.13, 123.30, 34.53, 27.10, 23.22, 22.79, 13.51. MS (ESI): m/z = (M + H) 198, Calculated value C_14_H_15_N 197.12.

**9-methyl-2,3-dihydro-1H-cyclopenta[b]quinoline:** Pale yellow oil, yield: 92 %, 168 mg, ^1^H NMR (400 MHz, CDCl_3_) δ 8.02 (d, *J* = 0.8 Hz, 1H), 8.00 (d, *J* = 0.8 Hz, 1H), 7.95 (d, *J* = 1.0 Hz, 1H), 7.93 (d, *J* = 1.1 Hz, 1H), 7.63 (d, *J* = 1.4 Hz, 1H), 7.61 (t, *J* = 1.4 Hz, 1H), 7.59 (d, *J* = 1.4 Hz, 1H), 7.50 (d, *J* = 1.3 Hz, 1H), 7.48 (t, *J* = 1.4 Hz, 1H), 7.46 (d, *J* = 1.3 Hz, 1H), 3.17 (t, *J* = 7.7 Hz, 5H), 3.06 (t, *J* = 7.4 Hz, 5H), 2.26 – 2.14 (m, 5H);  ^13^C NMR (101 MHz, CDCl_3_) δ 167.02, 147.49, 138.06, 134.03, 129.20, 127.99, 127.08, 125.24, 123.35, 35.14, 29.67, 22.84, 14.08. MS (ESI): m/z = (M + H) 184, Calculated value C_14_H_15_N 183.1.

**9-methyl-3,4-dihydroacridin-1(2H)-one:** Brown oil, yield: 91%, 192 mg, ^1^H NMR (400 MHz, CDCl_3_) δ 8.21 (dd, *J* = 8.3, 1.0 Hz, 1H), 8.01 (dd, *J* = 8.4, 0.7 Hz, 1H), 7.77 (ddd, *J* = 8.3, 6.8, 1.4 Hz, 1H), 7.57 (ddd, *J* = 8.3, 6.8, 1.3 Hz, 1H), 3.30 – 3.24 (m, 2H), 3.05 (s, 3H), 2.81 (t, *J* = 6.6 Hz, 2H), 2.26 – 2.15 (m, 2H);  ^13^C NMR (101 MHz, CDCl_3_) δ 200.64, 162.14, 149.95, 147.95, 131.50, 129.18, 127.72, 126.35, 125.48, 125.41, 53.43, 41.10, 34.79, 21.34, 16.06. MS (ESI): m/z = (M + H) 212, Calculated value C_14_H_15_N 211.09.

**6-chloro-4-(2-chlorophenyl)-2-phenylquinoline**: Off-white solid, yield: 81 %, 283 mg. ^1^H NMR (400 MHz, CDCl_3_) δ 8.14 – 8.11 (m, 1H), 8.10 (dd, *J* = 3.4, 2.2 Hz, 1H), 7.75 (s, 1H), 7.59 (dd, *J* = 9.0, 2.3 Hz, 1H), 7.53 (dd, *J* = 7.8, 1.5 Hz, 1H), 7.49 – 7.44 (m, 1H), 7.44 – 7.40 (m, 1H), 7.39 – 7.35 (m, 1H), 7.33 – 7.28 (m, 1H). ^13^C NMR (101 MHz, CDCl_3_) δ 157.03, 146.95, 145.77, 139.06, 136.33, 133.15, 132.37, 131.60, 131.35, 130.63, 130.14, 130.10, 129.68, 127.21, 126.54, 124.38, 120.60, 77.34, 77.02, 76.28. LCMS (ESI/TOF-Q): m/z = (M + H) 350.14, Calculated value C_21_H_13_Cl_2_N 349.04.

**1-(6-chloro-4-(2-chlorophenyl)-2-phenylquinolin-3-yl)ethan-1-one**: Pale brown solid, yield: 72 %, 277 mg, ^1^H NMR (400 MHz, CDCl_3_) δ 8.02 (d, *J* = 9.0 Hz, 1H), 7.62 (dd, *J* = 9.0, 2.3 Hz, 1H), 7.58 (d, *J* = 1.1 Hz, 1H), 7.56 (t, *J* = 1.6 Hz, 1H), 7.46 – 7.40 (m, 1H), 7.28 (s, 1H), 7.26 (s, 1H), 7.25 – 7.23 (m, 1H), 7.21 (dd, *J* = 6.3, 4.6 Hz, 1H), 7.20 – 7.18 (m, 1H), 7.18 (d, *J* = 2.0 Hz, 1H), 7.16 (t, *J* = 2.5 Hz, 1H), 7.15 (s, 1H), 7.12 (dd, *J* = 9.8, 4.3 Hz, 1H). ^13^C NMR (101 MHz, CDCl_3_) δ 197.02, 155.22, 145.87, 142.29, 136.46, 134.07, 133.76, 133.34, 132.90, 132.77, 132.06, 131.22, 130.67, 130.37, 129.66, 129.21, 128.55, 126.71, 125.56, 124.82, 31.38. LCMS (ESI/TOF-Q): m/z = (M + H) 392.16, Calculated value C_23_H_15_Cl_2_NO 291.05.

**(6-chloro-4-(2-chlorophenyl)-2-phenylquinolin-3-yl)(phenyl)methanone:** Off-white solid, yield: 64 %, 290 mg, ^1^H NMR (400 MHz, CDCl_3_) δ 8.16 (d, *J* = 9.0 Hz, 1H), 7.67 (dd, *J* = 9.0, 2.3 Hz, 1H), 7.58 – 7.53 (m, 2H), 7.44 (d, *J* = 7.6 Hz, 2H), 7.31 – 7.25 (m, 2H), 7.24 – 7.16 (m, 6H), 7.15 – 7.08 (m, 3H) ^13^C NMR (101 MHz, CDCl_3_) δ 196.52, 157.00, 146.17, 143.87, 139.51, 137.15, 133.50, 133.28, 131.59, 130.36, 129.64, 129.28, 128.98, 128.39, 128.11, 126.67, 125.99, 124.94, 121.9. LCMS (ESI/TOF-Q): m/z = (M + H) 454.18, Calculated value C_28_H_17_Cl_2_NO 453.06.
